# Novel Promising Antifungal Target Proteins for Conquering Invasive Fungal Infections

**DOI:** 10.3389/fmicb.2022.911322

**Published:** 2022-06-16

**Authors:** Cheng Zhen, Hui Lu, Yuanying Jiang

**Affiliations:** Department of Pharmacy, Shanghai Tenth People’s Hospital, School of Medicine, Tongji University, Shanghai, China

**Keywords:** antifungal targets, inhibitors, invasive fungal infections, drug resistance, drug toxicity

## Abstract

Invasive fungal infections (IFIs) pose a serious clinical problem, but the antifungal arsenal is limited and has many disadvantages, such as drug resistance and toxicity. Hence, there is an urgent need to develop antifungal compounds that target novel target proteins of pathogenic fungi for treating IFIs. This review provides a comprehensive summary of the biological functions of novel promising target proteins for treating IFIs in pathogenic fungi and their inhibitors. Inhibitors of inositol phosphoramide (IPC) synthases (such as Aureobasidin A, Khafrefungin, Galbonolide A, and Pleofungin A) have potent antifungal activities by inhibiting sphingolipid synthesis. Disrupting glycosylphosphatidylinositol (GPI) biosynthesis by Jawsamycin (an inhibitor of Spt14), M720 (an inhibitor of Mcd4), and APX001A (an inhibitor of Gwt1) is a promising strategy for treating IFIs. Turbinmicin is a natural-compound inhibitor of Sec14 and has extraordinary antifungal efficacy, broad-antifungal spectrum, low toxicity, and is a promising new compound for treating IFIs. CMLD013075 targets fungal heat shock protein 90 (Hsp90) and has remarkable antifungal efficacy. Olorofim, as an inhibitor of dihydrolactate dehydrogenase, is a breakthrough drug treatment for IFIs. These novel target proteins and their inhibitors may overcome the limitations of currently available antifungal drugs and improve patient outcomes in the treatment of IFIs.

## Introduction

Invasive fungal infections (IFIs) pose a severe clinical problem that causes approximately one and a half million deaths annually (Lee et al., 2021). Currently, there are only three frontline antifungal drugs for the treatment of IFIs: polyenes, echinocandins, and azoles, and each of them has disadvantages that limit their clinical application (Robbins et al., 2016). For example, polyenes have severe side effects, such as nephrotoxicity, due to the structural similarity between their target ergosterol and mammalian membrane sterol cholesterol (Perfect, 2017); Whereas echinocandins have a limited antifungal spectrum, a requirement for intravenous administration, and high drug costs (Perlin, 2015), Azoles have only fungistatic effects which result in the emergence of azole resistance (Zavrel and White, 2015). Therefore, uncovering alternative antifungal targets is necessary to expand the currently limited arsenal of antifungal agents for treating IFIs.

**Figure 1:**
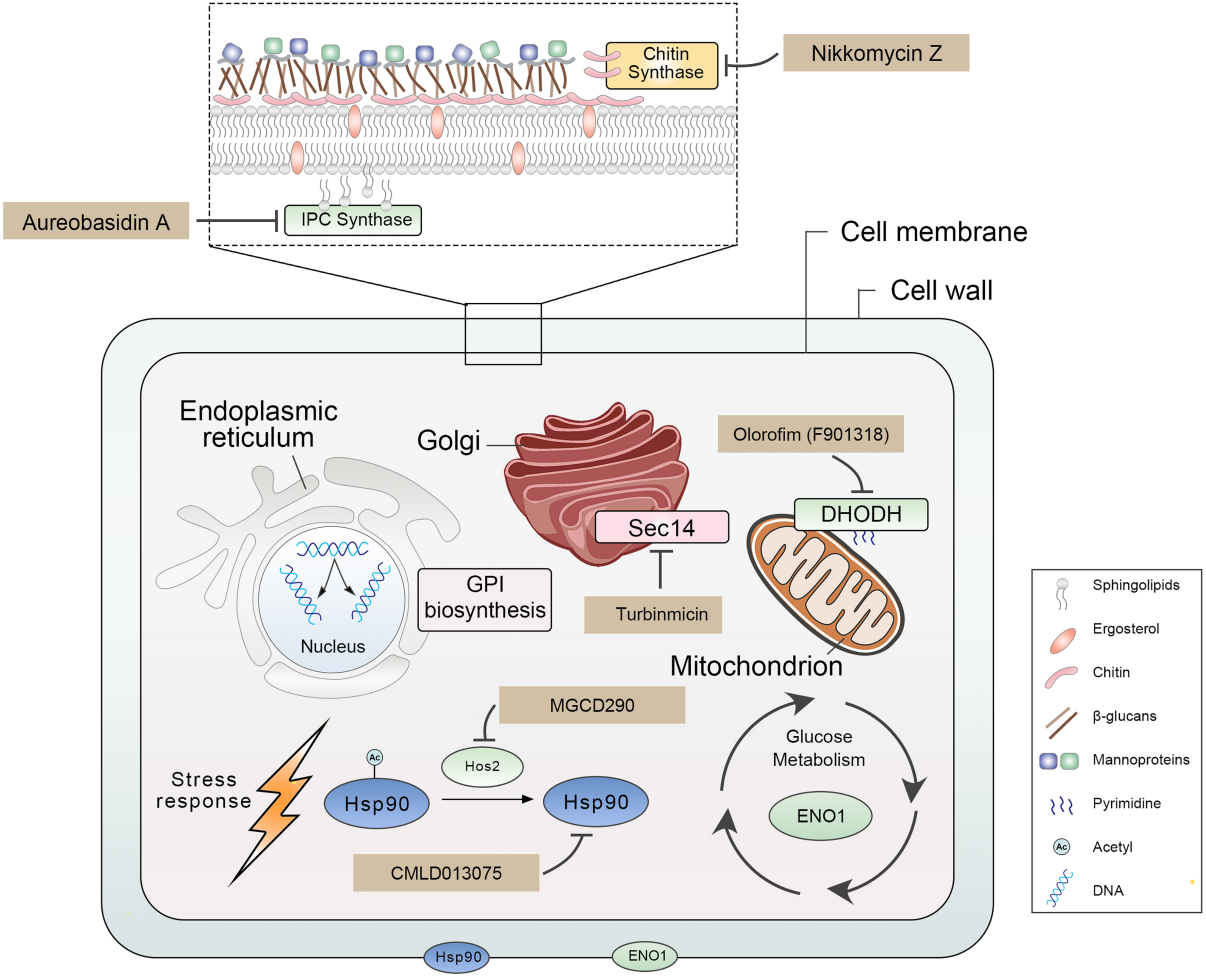
Overview of antifungal target proteins and their inhibitors.

We summarized nine promising antifungal target proteins and inhibitors for treating IFIs (Figure 1; Table 1). These target proteins meet the following criteria: (1) these proteins play a crucial role in the growth, virulence, and drug sensitivity of pathogenic fungi; (2) targeting these proteins is a new promising strategy for treating IFIs; (3) inhibitors of these proteins have been developed and have characteristics of remarkable antifungal efficacy, broad-antifungal spectrum, and low toxicity. Proteins included in this review play an important role in many biological processes of pathogenic fungi, suggesting that inhibiting these biological functions are promising new antifungal strategies. Firstly, damaging fungal cell wall integrity is an effective antifungal strategy, including disrupting glycosylphosphatidylinositol (GPI) synthesis by inhibiting Gwt1 (Umemura et al., 2003; Watanabe et al., 2012), Spt14 (Schönbächler et al., 1995; Fu et al., 2020), and Mcd4 (Maneesri et al., 2005; Mann et al., 2015) and inhibiting chitin synthase (Gaughran et al., 1994; Munro et al., 2001) decreased chitin, which is an essential part of the carbohydrate skeleton of the fungal cell wall. Secondly, the destruction of cell membrane integrity and membrane network trafficking is a promising antifungal strategy, such as targeting inositol phosphoramide (IPC) synthases suppressed sphingolipid synthesis (Heidler and Radding, 1995; Hashida-Okado et al., 1996) and inhibiting Sec14 blocked membrane trafficking from the Golgi network (Curwin et al., 2009; Zhang et al., 2020). Besides, heat shock protein 90 (Hsp90) plays an important role in pathogenic fungi’ survival, virulence, and drug resistance. Thus, impairing the function of Hsp90 contributes to treating IFIs by directly targeting Hsp90 (Whitesell et al., 2019; Hohrman et al., 2021) or preventing its acetylation by targeting histone deacetylase 2 (Hos2; Pfaller et al., 2009; Zacchi et al., 2010). Finally, disrupting glycolysis (targeting Eno1; Ko et al., 2013; Pitarch et al., 2014) and pyrimidine biosynthesis (targeting dihydrolactate dehydrogenase (DHODH; Zameitat et al., 2006; Oliver et al., 2016) are perspective antifungal strategies. Developing a new antifungal agent targeting new proteins helps expand the limited antifungal arsenal, curb the emergence of antifungal drug resistance, and improve patient outcomes in the treatment of IFIs.

**Table 1:** Novel promising targets and their inhibitors for the treatment of IFIs.

Target proteins	Target function	Inhibitors	Spectrum of activity	Development stage
**GPI biosynthesis**				
Spt14	Catalytic subunit of fungal UDP-glycosyltransferase, which is essential for GPI biosynthesis	Jawsamycin (FR-900848)	Broad spectrum	Preclinical
Mcd4	Transferring phosphoethanolamine groups (EtNP) during GPI biosynthesis	M743 (YW3548)	*Candida spp.* *Aspergilus* spp.	Preclinical
		M720	*Candida spp.* *Aspergilus spp.*	Preclinical
Gwt1	Transferring fatty acyl chains to the inositol fraction of GPI precursors	BIQ	*Candida albicans* *Microsporum spp.* *Trichophyton spp.*	Preclinical
		APX001	Broad spectrum	Phase I (NCT02957929-completed, NCT02956499-completed, NCT03333005-completed, NCT04166669-completed) Phase II (NCT03604705-completed, NCT04240886-planned)
**Chitin biosynthesis**				
Chitin synthases	Catalyzing the chitin extension chain, which plays an essential role in maintaining fungal cell wall integrity	Nikkomycin Z	*Candida albicans* *Candida auris* *Candida parapsilosis* (combine with fluconazole) *Cryptococcus neoformans* (combine with fluconazole) *A. fumigatu* (combine with caspofungin)	Phase I (NCT00834184-completed) Phase II (NCT00614666-terminated)
**Sphingolipids Biosynthesis**				
Inositol phosphoramide (IPC) synthase	Catalyzing the transfer of the phosphoinositol head group of phosphatidylinositol to the C1-hydroxyl group of ceramide to produce IPC	Aureobasidin A	Broad spectrum	Preclinical
		Khafrefungin	*Candida albicans* *Cryptococcus neoformans* *Saccharomyces cerevisiae*	Preclinical
		Galbonolide A	*Cryptococcus neoformans*	Preclinical
		Pleofungin A	*Candida spp.* *Cryptococcus neoformans* *Aspergilus spp.*	Preclinical
**Proteins membrane trafficking**				
Sec14	Transferring the phosphatidylinositol, plays an important role in regulating the interface between lipid metabolism and membrane trafficking from the Golgi network	Turbinmicin	Board spectrum	Preclinical
**Hsp90 function**				
Hsp90	Regulating the correct folding, transport, maturation, and degradation of client proteins	CMLD013075	*Candida albicans*	Preclinical
		Mycograb C28Y	*Candida spp.*	Preclinical
Histone deacetylase 2	Removing lysine residues from core histones, which control gene transcription and expression	MGCD290	Board spectrum	Phase II (NCT01497223-completed)
**Glycolysis**				
Eno1	Regutating the glycolysis pathway, plays a vital role in adhesion, hyphae formation, susceptibility to antifungal drugs, and virulence	MAb R-5	*Aspergilus spp.*	Preclinical
**Pyrimidine biosynthesis**				
Dihydrolactate dehydrogenase (DHODH)	Catalyzing the pyrimidine biosynthesis pathway	Olorofim (F901318)	*Aspergilus spp.* *Scedosporium* *Madurella mycetomatis* *Fusarium spp.*	Phase I (NCT03340597-completed, NCT02142153-completed, NCT02342574-completed, NCT02394483-completed, NCT02737371-completed) Phase II (NCT03583164-recruiting) Phase III (NCT05101187-not yet recruiting)

## Glycosylphosphatidylinositol Biosynthesis

**Figure 2:**
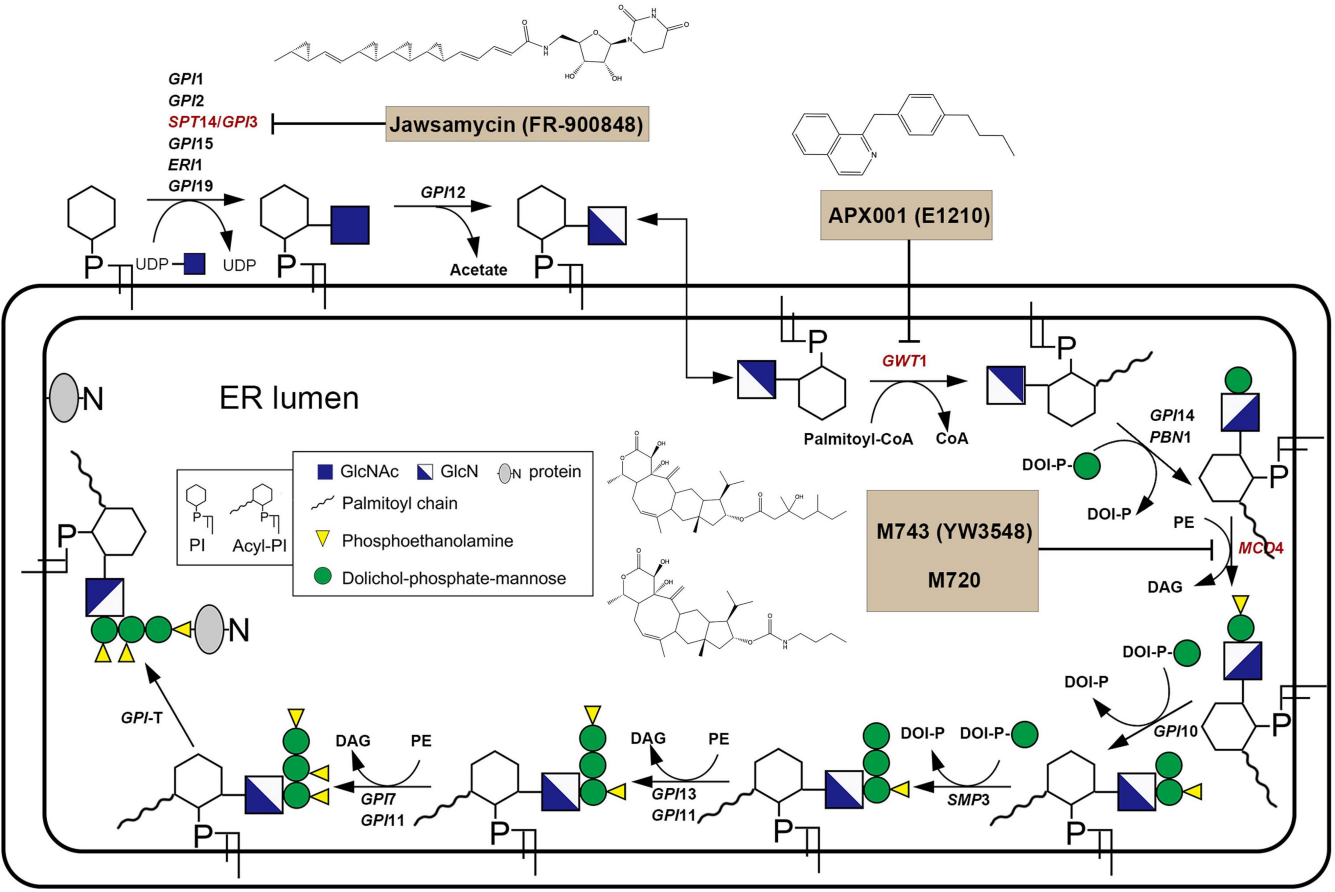
Overview of GPI biosynthesis in yeast.

In fungi, GPI plays an integral role in anchoring proteins to the plasma and is thus critical to the fungal cell wall integrity (Yadav and Khan, 2018; Fu et al., 2020). GPI biosynthesis starts from the cytoplasmic side of the endoplasmic reticulum (ER). That is, N-acetylglucosamine (GlcNAc) is transferred from UDP-GlcNAc to phosphatidylinositol (PI), forming N-acetyl glucosaminyl phosphatidylinositol (GlcNAc-PI). This reaction is catalyzed by the GPI-GlcNAc transferase (GPI-GnT) complex comprising six core subunits. In the GPI-GnT complex, UDP-glycosyltransferase catalytic subunit (Spt14) acts as the catalytic subunit. GlcNAc-PI is then de-N-acetylated to glucosaminyl-phosphatidylinositol (GlcN-PI). GlcN-PI is acylated by the acyl-Coa-dependent inositol acyltransferase (Gwt1), generating GlcN-(acyl)PI. First and second mannoses are then added sequentially to the GlcN-(acyl)PI by the GPI-α1,4-mannosyltransferase-I (Gpi14/Pbn1) and GPI-α1,6-mannosyltransferase-II (Gpi18/Pga1), respectively, leading to the formation of Man–Man-GlcN-(acyl)PI intermediate. And then phosphoethanolamine group (EtNP) is transferred from Man–Man-GlcN-(acyl)PI to the 2-position of first mannose by the GPI-phosphoethanolamine transferase-I (Mcd4) generating Man-(EtNP)Man-GlcN-(acyl)PI. Third and fourth mannose additions carry out by the GPI-α1,2-mannosyltransferase-III (Gpi10) and GPI-α1,2-mannosyltransferase-IV (Smp3), respectively, resulting in the formation of Man-Man-Man-(EtNP)Man-GlcN-(acyl)PI. Phosphoethanolamine moieties are then added to the 6-position of third and second mannose, respectively, by the GPI-phosphoethanolamine transferase-III (Gpi13/Gpi11) and GPI-phosphoethanolamine transferase-II (Gpi7/Gpi11), resulting in the formation of complete GPI precursor, Man-(EtNP)Man-(EtNP)Man-(EtNP)Man-GlcN-(acyl)PI (Figure 2; Yadav and Khan, 2018).

### Spt14

The fungal UDP-glycosyltransferase catalytic subunit, also known as Spt14, encoded by the *SPT14* gene (YPL175W) in *Saccharomyces cerevisiae* is essential for GPI biosynthesis. The homozygous deletion of the *SPT14* gene resulted in defective GPI anchoring due to a defect in the synthesis of GlcNAc-PI, the first step of GPI synthesis (Yadav and Khan, 2018). The *SPT14* gene (CR_04040C_A) is vital for *Candida albicans* because the loss of the *SPT14* gene led to this pathogenic fungus being inviable (Nobile and Mitchell, 2005; Segal et al., 2018). In *Aspergillus fumigatus*, the loss of *the afpig-a* gene, the homolog of the *SPT14* gene, resulted in complete blocking of the GPI synthesis and led to cell wall defect, abnormal hyphal growth, rapid growth conidial germination, and aberrant conidiation (Li et al., 2007).

Jawsamycin (FR-900848) is a natural product containing oligomeric cyclopropyl (Figure 2). Genetic follow-up and unbiased resistance profiling studies pointed to the catalytic subunit Spt14 as the target of jawsamycin (Fu et al., 2020). Although the *S. cerevisiae* Spt14 shares 40% identity with human homolog PIG-A (Table 2), jawsamycin selectively targets fungal Spt14 but not the human PIG-A (Fu et al., 2020). Jawsamycin exhibited antifungal activity with broad-spectrum and potency against *Fusarium* species, *Scedosporium* species, and *Mucorales* fungi, including *Rhizopus oryzae* and *Absidia corymbifera*, and *Mucor circinelloides* (Fu et al., 2020). It is worth noting that these fungal species are generally insensitive to current licensed antifungal agents (Yoshida et al., 1990). Besides, jawsamycin has also demonstrated antifungal activity in a mouse model of invasive pulmonary mucormycosis caused by *Rhyzopus delemar* (Fu et al., 2020). Notably, jawsamycin has low toxicity to mice at the tested doses (Fu et al., 2020). In summary, jawsamycin, as an inhibitor of Spt14, is a promising antifungal agent with a broad-antifungal spectrum and low toxicity.

**Table 2:** Comparison of amino acid consistency of target proteins between fungal and mammalian.

Target proteins	Fungal-unique	Mammalian homolog	The proportion of amino acid sequence identity
Spt14	No	PIG-A	40% (*S. cerevisiae*)
Gwt1	No	PIG-W	30% (*S. cerevisiae*)
Mcd4	No	PIG-N	36% (*S. cerevisiae*)
Chitin synthases	Yes	–	–
Sec14	No	SEC14-like protein 1	11% (*S. cerevisiae*)
IPC synthase	Yes	–	–
Hsp90	No	HSP 90-alpha	60% (*S. cerevisiae*)
		HSP 90-beta	61% (*S. cerevisiae*)
Histone deacetylase 2	Yes	–	–
Eno1	No	Alpha-enolase	63% (*S. cerevisiae*)
Dihydrolactate dehydrogenase	No	Dihydroorotate dehydrogenase	30% (*Aspergillus*)

### Gwt1

Gwt1 is a catalytic inositol acyltransferase that transfers fatty acyl chains to the inositol fraction of GPI precursors. Gwt1 plays a crucial role in maintaining fungal cell wall integrity and enabling cell adhesion to mucosal surfaces. The newly isolated temperature-sensitive *gwt1* (YJL091C) mutants are defective in cell wall biosynthesis, leading to the defect (Umemura et al., 2003). Besides, the *gwt1* mutant was delayed in the ER-Golgi transport of the GPI-anchored protein, Gas1, and drastically reduced the incorporation of radiolabeled inositol into proteins. This phenomenon may be due to the defects of GPI transfer to protein and defective inositol acylation in GPI biosynthesis (Umemura et al., 2003). In *C. albicans*, the *GWT1/gwt1*Δ mutant showed reduced growth rate, hyphal development, and virulence in mice. A *gwt1*∆*/gwt1*∆ null mutant was inviable, indicating that the *GWT1* gene is essential for *C. albicans* (Watanabe et al., 2012). Besides, Gwt1 shows a low degree of identity to the closest direct mammalian homolog (<30% amino acid sequence identity; Table 2), making it an excellent target for the design of novel antifungal agents (Umemura et al., 2003; Yadav and Khan, 2018). However, elucidation of the 3D structure of the target is lacking because Gwt1 has an estimated 13 transmembrane structural domains (Sagane et al., 2011).

APX001A (formerly E1210, Amplyx; Figure 2) is optimized from 1-[4-butylbenzyl] isoquinoline (BIQ; Tsukahara et al., 2003) and has strong selectivity for the fungal Gwt1 (Watanabe et al., 2012). It has broad-spectrum antifungal activity with low MIC values against *Candida tropicalis*, *Candida glabrata*, *C. glabrata*, *C. auris*, and echinocandin-resistant *C. glabrata* (Miyazaki et al., 2011; Watanabe et al., 2012). In animal models, APX001A acts significantly effective against IFIs caused by the azoles-resistant *C. albicans*, *C. tropicalis*, *Aspergillus flavus* (in combination with voriconazole or caspofungin), and *A. fumigatus*. Besides, the prodrug APX001 was evaluated in an immunocompromised murine model of disseminated *C. auris* infection. Significant efficacy was observed in all three APX001 treatment groups versus 50% survival for the anidulafungin treatment group (Hata et al., 2011; Berkow and Lockhart, 2018; Pfaller et al., 2019; Wiederhold et al., 2019). There are four phases I clinical trials to examine the safety and tolerability of APX001 (Table 1). A phase I trial evaluated six single ascending dose (SAD) and four multiple ascending doses (MAD) cohorts, in which subjects were randomized in a 6:2 ratio to receive 3 h of intravenous infusions of APX001 or placebo (ClinicalTrials.gov Identifiers: NCT02956499). SAD cohorts received doses from 10 to 350 mg, whereas MAD cohorts received 50–600 mg once daily for 14 days. APX001 was well-tolerated across all doses with no clinically significant adverse events observed, and there were no dose-limiting toxicities. Most of the adverse effects (AEs) were mild, transient, and required no treatment, with the most common AE being headache (Hodges et al., 2017b). Another phase I trial gauged the safety, pharmacokinetics, bioavailability, and food effects of orally administered fosmanogepix (ClinicalTrials.gov Identifiers: NCT02957929). Patients in this trial were randomized to single intravenous doses of 200 mg infused over 3 h followed by single oral dosing (tablet) of 100, 300, and 500 mg, each separated by a 14-day washout period. Subjects were also evaluated under fed and fasting conditions following a single oral dose of 400 mg. This phase I trial suggested that APX001A was well tolerated across all studied doses, with no clinically significant AEs observed (Hodges et al., 2017a). A clinical phase II trial completed in 2021 evaluated the efficacy and safety of prodrug APX001 for the first-line treatment for candidemia, including suspected or confirmed antifungal-resistant candidemia in non-neutropenic patients (ClinicalTrials.gov Identifiers: NCT03604705). The results showed that, among the 20 participants, 80% of patients with eradicative mycological outcomes at the end of study treatment, one patient recurrent in follow-up 2 weeks after End of Antifungal Treatment (EOT), and none of the patients recurrent in follow-up 4 weeks after EOT. However, results also show that 95.2% of patients had reported adverse events such as gastrointestinal disorders, edema peripheral, and pyrexia. Besides, another clinical phase II trial evaluating APX001 in the treatment of IFIs caused by *Aspergillus* species or rare mycobacteria such as *Scedosporium* species, *Fusarium* species, and *Mucorales* is currently underway (ClinicalTrials.gov Identifiers: NCT04240886). These results suggest that disrupting GPI synthesis is a practical strategy for treating IFIs.

### Mcd4

Mcd4 is a phosphoethanolamine transferase that plays a role in transferring phosphoethanolamine groups (EtNP) during GPI biosynthesis (Pittet and Conzelmann, 2007; Yadav and Khan, 2018). It is a transmembrane protein located within the ER (Gaynor et al., 1999). Mcd4 is essential for synthesizing the GPI core structure of *S. cerevisiae*, and the *MCD4* gene deletion mutant growth is slow (Maneesri et al., 2005). The *MCD4* gene deletion mutant showed a decrease in GPI cell wall protein levels, a decline in mannan levels, and an increase in alkali-insoluble β-1,6-glucan and chitin levels in the cell wall (Maneesri et al., 2005). Similarly, the Mcd4 also plays a vital role in GPI synthesis in *C. albicans*. Repression of the *MCD4* gene expression led to a decrease in growth and abnormal morphology in *C. albicans* (Hasegawa et al., 2019). Besides, fungal Mcd4 has a low overall identity (36%) compared to human homolog PIG-N (Table 2).

M743 (YW3548; Figure 2) is an inhibitor of Mcd4 and has potent antifungal activity by inhibiting phosphoethanolamine-modified mannose (Peter and Menon, 2007). It has antifungal activity against various pathogenic fungi, including *C. albicans*, *C. parapsilosis*, *Candida glabrata*, *Candida krusei*, *C. lusitaniae*, and *A. fumigatus* (Mann et al., 2015). However, the stability of M743 is poor, and it is quickly inactivated by hydrolysis. Thus, the M743 esterase sensitive linkage was replaced with a carbamate linkage, yielding the compound M720 (Figure 2), which displayed highly favorable stability in mouse plasma without any loss in antifungal activity. Although M720 has cytotoxicity in various human cell lines, M720 remains highly selective to its cognate target in an animal model of candidiasis (Mann et al., 2015). Besides, inhibitors of Gwt1 and Mcd4 have sound synergistic antifungal effects (Mann et al., 2015). These results suggested that disrupting GPI biosynthesis is beneficial to treating IFIs.

## Chitin Synthase

Chitin synthases are members of the GT-2 glycosyltransferase family and catalyze the chitin extension chain, essential in maintaining fungal cell wall integrity. They contain multiple transmembrane structural domains and require divalent metal ions (usually Mg^2+^) to be active (Dorfmueller et al., 2014; Morozov and Likhoshway, 2016). Chitin synthases are present as isoenzymes in fungi; the number varies from three in *S. cerevisiae*, four in *C. albicans* to eight in *A. fumigatus* and *Aspergillus nidulans* (Yang and Zhang, 2019). Based on amino acid sequence similarities, seven chitin synthases have been identified. Four chitin synthases of *C. albicans* are two class I enzymes (Chs2 and Chs8), one class II enzyme (Chs1), and one class IV (Chs3) enzyme. Chs1 synthesizes the primary septal chitin, contributes to general cell wall integrity, and is the only known chitin synthase essential for growth (Munro et al., 2001). The class I enzyme Chs2 is not critical for growth, and loss of Chs2 did not lead to significantly attenuated virulence of *C. albicans* in normal and immunosuppressed mice (Gow et al., 1994). Similarly, lacking the other class I enzyme, Chs8, did not affect growth rates, cellular morphologies, and chitin contents of *C. albicans*. However, the chitin content of the *chs2*Δ/∆*chs8*Δ/∆ double mutant decreased by more than 97% (Lenardon et al., 2009). Chs3 is required to synthesize the chitin rings found on the surface of yeast cells. Despite the homozygous deletion of the *CHS3* gene mutants having regular growth rates *in vitro*, the mutants are significantly less virulent than the parental strain in immunocompetent and immunosuppressed mice (Bulawa et al., 1995). Characterization of each chitin synthase activity is less understood in molds than in yeast because of the higher number of genes in filamentous fungi and the possibility of masking a mutation by over-or alternate expression of different *CHS* genes. Eight chitin synthases of *A. fumigatus* are one class I enzyme (ChsA), one class II enzyme (ChsB), two class III enzymes (ChsC, ChsG), one class IV enzyme (ChsF), one class V (ChsE), one class VI (ChsH), and one class VII enzyme (ChsD; Yang and Zhang, 2019). In *A. fumigatus* chitin synthase family, *chsG* and *chsE*, play a role in the morphogenesis of this fungal species. An *A. fumigatus* strain lacking both *chsG* and *chsE* genes has reduced chitin synthase activity, has reduced colony radial growth rate, produces highly branched hyphae, and shows alterations in the shape and germination capacity of the conidia (Mellado et al., 2003). It is worth noting that disruption of chitin synthesis is a potential strategy for the treatment of IFIs due to this synthesis being fungal-specific.

**Figure 3:**
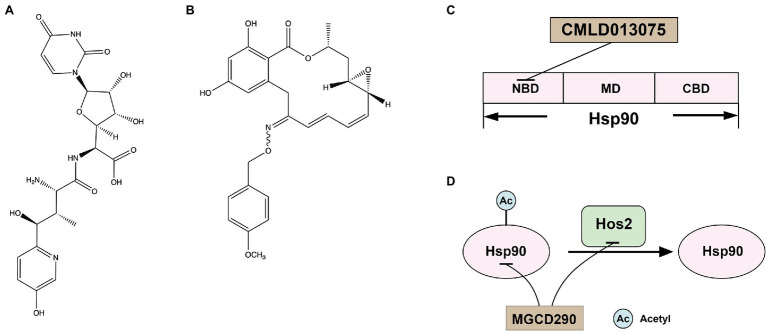
**(A)** Structure of chitin synthase inhibitor Nikkomycin Z; **(B)** structure of Hsp90 inhibitor CMLD013075; **(C)** the docking model of CMLD013075 and Hsp90; and **(D)** the interaction of Hsp90 and Hos2.

Nikkomycin Z (Figure 3A), a competitive inhibitor of chitin synthase, was first discovered by Bayer in the 1970s (Fiedler et al., 1982). Nikkomycin Z inhibited Chs I and Chs III, but not ChsII, of *S. cerevisiae* and inhibited all Chs isozymes of *C. albicans* (Gaughran et al., 1994). Nikkomycin Z inhibited the growth of *C. albicans* but showed low antifungal activity against *A. fumigatus* (Fortwendel et al., 2009). In addition, nikkomycin Z and caspofungin had synergistic antifungal activity against both *A. fumigatus* and *C. albicans* (Fiedler et al., 1982; Walker et al., 2008; Fortwendel et al., 2009). Similarly, nikkomycin Z enhanced the antifungal activities of fluconazole and itraconazole against *C. albicans*, *C. parapsilosis*, *Cryptococcus neoformans*, and *Coccidioides immitis* (Li and Rinaldi, 1999). A recent study demonstrated that Nikkomycin Z also had a tremendous antifungal activity against *C. auris* (Bentz et al., 2021). Nikkomycin Z has completed INDA (Investigational New Drug Application) and clinical phase I trials (ClinicalTrials.gov Identifiers: NCT00834184). It was absorbed after oral administration, reaching a maximum concentration in serum of 2.21 μg/ml at 2 h postdose. No severe or dose-related adverse events were observed and showed excellent safety in healthy humans (Nix et al., 2009). A clinical phase II trial (ClinicalTrials.gov Identifiers: NCT00614666) to determine a safe dose in patients with pulmonary coccidioidomycosis was terminated early due to recruitment challenges and lack of funding, but a clinical phase II A trial is planned (Larwood, 2020).

## Inositol Phosphoramide Synthase

In pathogenic fungi, sphingolipids consist of IPC and their mannosylated derivatives, are essential components of eukaryotic biological membranes (van der Rest et al., 1995; Dickson, 1998). IPC synthase acts as an inositol phosphoryl transferase, catalyzing the transfer of a phosphoinositol head group from phosphatidylinositol to ceramide to produce IPC (Levine et al., 2000). The *AUR1* gene (YKL004W), which encodes IPC synthase, is essential for *S. cerevisiae* survival because the homozygous deletion of the *AUR1* gene makes *S. cerevisiae* cells inviable (Heidler and Radding, 1995; Hashida-Okado et al., 1996; Giaever et al., 2002). Similarly, *in vivo* transposon mutagenesis of the *AUR1* gene (C5_01240W_A) led to the death of *C. albicans* (Segal et al., 2018). Moreover, the heterozygous deletion of the *AUR1* gene results in decreased virulence of *C. albicans* (Becker et al., 2010). In *C. neoformans*, the conditional repression of the *IPC1* gene (encoding IPC synthase) resulted in reduced growth under acidic conditions (Luberto et al., 2001). The mutant with the *aurA* (a functional homolog of the *S. cerevisiae AUR1* gene) open reading frame (ORF) was disrupted in *A. nidulans* was accompanied by growth defects in spores and germlings, which were unable to establish a normal polarity axis (Cheng et al., 2001), suggesting that the *aurA* gene is essential for *A. nidulans* growth. Therefore, the IPC synthase is necessary for fungal growth and a potential antifungal target.

**Figure 4:**
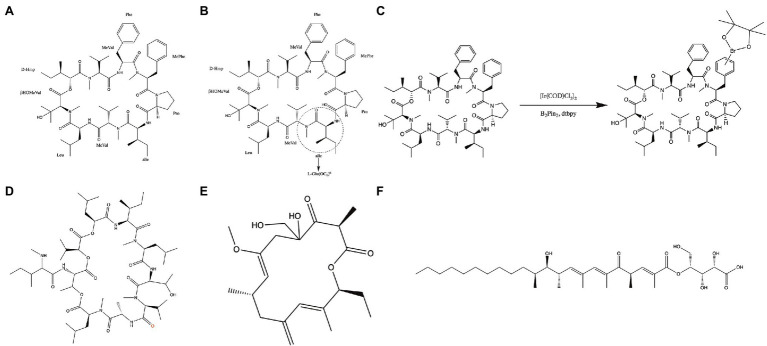
Inhibitors of the IPC synthase. **(A)** Structure of Aureobasidin A; **(B)** structure of [L-Glu(OC_6_)^6^]-AbA; **(C)** functionalization of the phenylalanine residues in the compound by iridium-catalyzed borylation; **(D)** structure of Pleofungin A; **(E)** structure of galbonolide A; and **(F)** structure of khafrefungin.

Aureobasidin A (AbA) is a natural molecular inhibitor of IPC synthase, which is a cyclic peptide comprised of a hydroxy acid and eight amino acids (Figure 4A; Kuroda et al., 1999; Xu et al., 2007). AbA inhibits the IPC synthase at nanomolar concentrations and shows antifungal activity against *Candida*, *Cryptococcus*, and *Aspergillus* species with low toxicity (Takesako et al., 1993; Nagiec et al., 1997; Teymuri et al., 2021). Its antifungal activity for the most pathogenic fungi was superior to that of fluconazole and amphotericin B *in vitro* (Takesako et al., 1993; Aeed Paul et al., 2009; Teymuri et al., 2021). Besides, AbA can inhibit filamentation and biofilm development of *C. albicans* (Tan and Tay, 2013; Munusamy et al., 2018). Structure–activity relationship studies have demonstrated that modifying and exchanging the amino acid sequence of AbA can affect its pharmacological properties (Aeed Paul et al., 2009) and, consequently, its antifungal activity. Among the lipophilic analogs replaced at positions 6 of AbA with L-glutamic acid, the compound with hexyl ester group (Figure 4B) showed the most potent antifungal activity against *Candida* species (Kurome et al., 1998). Furthermore, functional phenylalanine residues in AbA by iridium-catalyzed borylation (Figure 4C) can improve the antifungal activity of AbA against *A. fumigatus* (Wuts et al., 2015). Khafrefungin, as one of the AbA derivatives, is a 22-carbon linear polyketide esterified to the C-4 hydroxyl of an aldonic acid, including four chiral centers (Figure 4F; Wakabayashi et al., 2001). The inhibitory effect of khafrefungin on IPC synthesis is due to the highly hydroxylated acidic polar headgroup on khafrefungin that resembles phosphoinositol (Mandala et al., 1997; Nakamura et al., 2003). Khafrefungin suppresses the growth of fungi such as *S. cerevisiae*, *C. albicans*, and *C. neoformans* at picomolar to nanomolar concentrations (Mandala et al., 1997). Galbonolide A (rustmicin) is also one of the AbA derivatives and a 14-membered macrolide (Figure 4E) with fungicidal activity against clinically essential strains (Mandala et al., 1998). It is incredibly potent against *C. neoformans*, inhibiting growth and sphingolipid synthesis at concentrations below 1 ng/ml (Mandala et al., 1998). In addition to AbA derivatives, pleofungins A, B, C, and D have been found from a mycelial extract of *Phoma* sp. and have a potent inhibitory effect on IPC synthase. Pleofungin A is a 28-membered cyclic depsipeptide consisting of eight amino acids and two 2-hydroxycarboxylic acids (Figure 4D), inhibiting the IPC synthase of *S. cerevisiae* and *A. fumigatus* at the half inhibitory concentration (IC_50_) values of 16 and 1 ng/ml (Yano et al., 2007). This inhibitor also suppressed the growth of *C. albicans*, *C. neoformans*, and *A. fumigatus* at minimum inhibitory concentration (MIC) values of 2, 0.3, and 0.5 μg/ml, respectively (Yano et al., 2007). Taken together, the IPC synthase plays a pivotal role in fungal membrane synthesis and is a plausible target for the treatment of IFIs (Nagiec et al., 1997; Nicola et al., 2019). And, the inhibitors targeting IPC synthase are promising molecules for treating IFIs.

## Sec14

Sec14, the major phosphatidylinositol-transfer protein (PITP), regulates lipid metabolism and membrane trafficking from the Golgi network. In the *sec14* null mutant of *S. cerevisiae*, the secretion of reverse transcriptase has been dramatically blocked (Bankaitis et al., 1989). The *SEC14* gene in *C. albicans* is essential for vegetative growth (Monteoliva et al., 1996). Sec14 fold is structurally conserved, comprising about 280-residue two-lobed globular structure. The two lobes of Sec14 from *S. cerevisiae* are formed by four antiparallel β-strands, bordered by two long α-helices, and the larger lobe has a phospholipid-binding pocket. Its topological structure is similar to a fist, with four fingers close to the bound phospholipid. In contrast, the opposite thumb is a small N-terminal lobe, which can close the phospholipid-binding pocket when Sec14p is bound to the ligand. In addition, the N-terminal portion of the conserved region is often annotated as a separate entity, called the CRAL_TRIO_N domain (PF03765). The phospholipid-binding pocket of Sec14 contains two molecules of octyl glucoside, which are essential for obtaining crystals (Saito et al., 2007; Bankaitis et al., 2010). Besides, Sec14 has a low degree of identity compared to human homolog SEC14-like protein 1 (11% amino acid sequence identity; Table 2), making it an excellent target for the design of novel antifungal agents.

**Figure 5:**
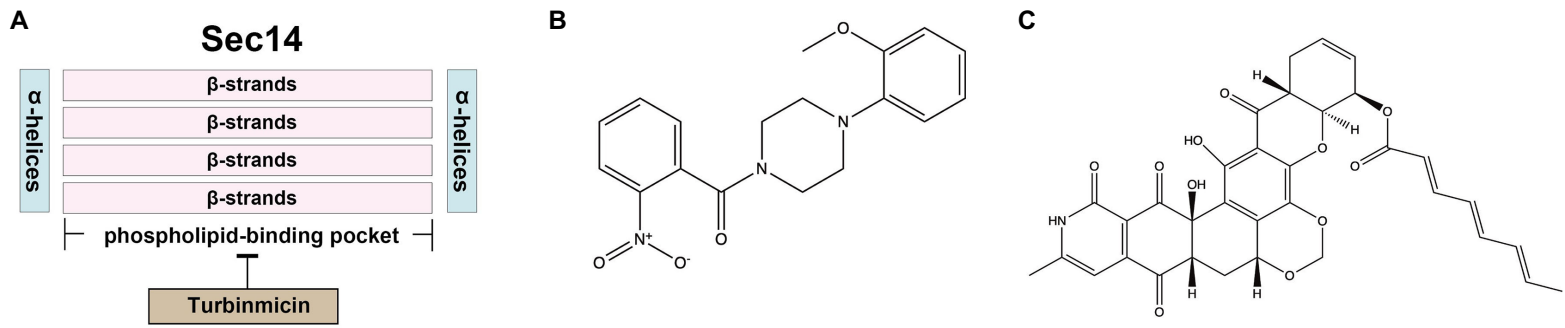
**(A)** The docking model of Turbinmicin and Sec14; **(B)** structure of Sec14 inhibitors NPPMs; and **(C)** structure of Sec14 inhibitor Turbinmicin.

Nitrophenyl[4-(2-methoxyphenyl)piperazin-1-yl] methanones (NPPMs) are small-molecule inhibitors of Sec14 (Figure 5B) and have antifungal activities *in vitro* and *in vivo* (Nile et al., 2014). A promising Sec14 inhibitor, turbinmicin, from a sea squirt microbiome constituent, *Micromonospora* species, had inspiring antifungal activity. Turbinmicin (Figure 5C) is a highly oxidized type II polyketide (Zhang et al., 2020) that can dock into the phospholipid-binding pocket of Sec14 (Figure 5A). Its heptacyclic ring system overlaps the co-crystallized ligand positions of picolinamide (6F0E) and octyl glucoside. Its polyene tail extends into a hydrophobic cleft left vacant by the co-crystallized ligands, producing a predominant binding mode. This compound has a broad-antifungal spectrum, including *C. albicans*, *C. auris*, *C. glabrata*, *C. tropicalis*, *A. fumigatus*, *Fusarium* species, and *Scedosporium* species, and *Rhizopus* species (Zhang et al., 2020). For most clinical isolates, the MIC of Turbinmicin was much lower than frontline antifungal drugs, such as azoles, polyenes, and echinocandins (Zhang et al., 2020). Moreover, in the preliminary safety study of human red blood cells, when the concentration of Turbinmicin is 1,000 times MIC, it does not trigger hemolysis and has high safety (Zhang et al., 2020). Besides, fungicidal activity can be observed at concentrations exceeding the MIC, much better than fluconazole. Turbinmicin can destroy the integrity of biofilm by destroying vesicle transport and subsequent assembly of the biofilm matrix. This property is also conducive to combining other antifungal drugs blocked by biofilm and cannot bind to the target and play an antifungal effect (Zhao et al., 2020). Turbinmicin has extraordinary antifungal efficacy, broad-antifungal spectrum, disrupting biofilm integrity, and low toxicity, and is a promising new compound for treating IFIs.

## Hsp90

Hsp90 is an essential molecular chaperone in eukaryotes, regulating the correct folding, transport, maturation, and degradation of client proteins. Hsp90 can modulate azole resistance by acting on calcineurin and Mkc1 kinase in *C. albicans* (LaFayette et al., 2010). Besides, Hsp90 also regulates mycelium formation, which is critical for virulence. In addition, in *C. albicans*, Hsp90 depletion reduced matrix glucan levels, leading to decreased azoles resistance to biofilm (Robbins et al., 2011). Hsp90 affects multiple pathogens’ survival, virulence, and drug resistance and may serve as a novel strategy to combat the escalating threat posed by drug-resistant fungi. Hsp90 has a conserved structure of homodimers. The N-terminal structural domain of Hsp90 is responsible for ATP binding, and the intermediate structural domain complements the nucleotide-binding site and binds client proteins. The C-terminal structural domain of Hsp90 is dimerization (Blacklock and Verkhivker, 2014). Although the crystal structure of fungal Hsp90 has been determined (Whitesell et al., 2019), the highly conserved amino acid sequence of Hsp90 in eukaryotes limited inhibitors of Hsp90 for treating IFIs. Whitesell L and his colleagues found that although Hsp90 is highly conserved, the nucleotide-binding domains (NBD) of Hsp90 are conformational flexibility. Encouraged by the conformational flexibility revealed by the NBD of *C. albicans* Hsp90, Whitesell L and his colleagues synthesized CMLD013075 (Figure 3B), which was more than 25-fold binding selectivity for fungal Hsp90 (Figure 3C; Whitesell et al., 2019; Huang et al., 2020). Therefore, CMLD013075 had low toxicity to mammalian cell lines compared to other Hsp90 inhibitors. CMLD013075 inhibited the growth of *C. albicans* and could enhance the antifungal effects of azole antifungals against resistant clinical isolates of *C. albicans* (Whitesell et al., 2019). CMLD013075, as a highly selective fungal Hsp90 inhibitor, is a successful example of the treatment of IFIs by inhibiting the function of fungal Hsp90.

Intracellular fungal Hsp90 can invade outside of fungal cells and become extracellular Hsp90 (eHsp90), located in the plasma, cell wall, and out of the fungal cells (Matthews and Burnie, 1992). In *A. fumigatus*, eHsp90 plays a crucial role in maintaining cell wall integrity (Lamoth et al., 2012). Besides, fungal eHsp90 can bind to various human serum proteins, rendering these proteins inoperative by affecting protein folding or interaction (Matthews and Burnie, 1992). Antibodies targeting eHsp90 have antifungal activities (Matthews et al., 2003), indicating that eHsp90 is a critical virulence factor of pathogenic fungi. Novartis mimicked endogenous antibodies and developed a recombinant monoclonal antibody, Mycograb, a 28 kDa human recombinant antibody fragment that binds to fungal Hsp90. Mycograb binds the middle domain of Hsp90, inhibiting communication between the terminal domains with client proteins (Bugli et al., 2013). Mycograb and amphotericin B had synergistic antifungal activity against *Candida* species *in vitro* and *in vivo* (Matthews et al., 2003). Besides, Mycograb enhanced the antifungal activity of fluconazole against *Candida* species *in vitro* (Nooney et al., 2007). However, unfortunately, due to the autoaggregation of Mycograb, the Committee for Medicinal Products for Human Use (CHMP) refused the marketing authorization in 2007. To meet the approval of CHMP, Novartis modified the structure of Mycograb by replacing the cysteine at position 28 with tyrosine to obtain Mycograb C28Y, which overcame the problem of spontaneous aggregation (Bugli et al., 2013). Unfortunately, unlike Mycograb, Mycograb C28Y does not synergize with amphotericin B *in vivo* (Louie et al., 2011).

## Histone Deacetylase 2

Histone deacetylases (HDACs) catalyze the removal of acetyl groups, leading to chromatin condensation (Thiagalingam et al., 2003). Therefore, these enzymes play crucial roles in regulating gene expression as they modulate the accessibility of chromatin to transcriptional regulators and other regulating factors (Thiagalingam et al., 2003). In mammalian cells, it has become appreciated that Hsp90 is subject to complex regulation by posttranslational modification. Histone deacetylase 2 (Hos2) regulates Hsp90 deacetylation (Figure 3D), which is required for Hsp90 interaction with select cochaperones and stabilization of several Hsp90 client proteins (Scroggins et al., 2007; Zacchi et al., 2010; Perfect, 2017; Van Daele et al., 2019). For example, *C. albicans* Hsp90 contains two acetylation sites, lysine 30 and lysine 271, which are not acetylated and will affect the function of Hsp90 (Li et al., 2017). In *S. cerevisiae* and other yeast, Hos2 and Set3 are parts of a similar multiprotein complex (Set3C) that possesses histone deacetylase activity (Pijnappel et al., 2001). In *C. albicans*, the deletions of essential subunits of Set3C display a hyper filamentous phenotype at elevated temperatures, specifically in the opaque phase (Hnisz et al., 2010). Besides, the *set3*Δ/Δ mutant and *hos2*Δ/Δ mutant cells also show strongly attenuated virulence in a murine model of systemic infection, which is associated with hyper filamentation *in vivo* (Hnisz et al., 2010). In *C. neoformans*, the deletion of the *HOS2* gene (CNAG_05563) resulted in growth defects at 37°C and a temporary delay in melanin production (Liu et al., 2008). The *hos2*Δ strains display compromised secreted protease activities (Brandão et al., 2018). The production of secreted proteases is an important virulence trait for *C. neoformans*, as they are involved in the growth and survival in the presence of antifungal drugs and the invasion of the central nervous system (Bien et al., 2009; Vu et al., 2014). Taken together, Hos2 has the potential to be an antifungal target.

MGCD290 (developed by MethylGene, Inc., Montreal, Canada) is an inhibitor of fungal Hos2. In addition to inhibiting the deacetylation of histone proteins, MGCD290 also inhibits the deacetylation of nonhistone proteins such as Hsp90 (Pfaller et al., 2015). Although MGCD290 only has modest activity against *Candida* species (Pfaller et al., 2015), it has good synergistic antifungal activity with azoles against many multi-drug resistance strains of *Candida* species *in vitro* (Pfaller et al., 2009). Besides, MGCD290 enhanced the antifungal activity of echinocandins against the echinocandin-resistant *Candida* species. Therefore, MGCD290 can be used as an adjuvant to enhance the antifungal activity of antifungal drugs against drug-resistance pathogenic fungi. However, MGCD290 combined with fluconazole in clinical trials was not more effective in treating moderate to severe vaginal candidiasis than fluconazole alone (ClinicalTrials.gov identifier: NCT01497223; Perfect, 2017). There are various reasons for this outcome, including different pharmacokinetics, fungal burden at the site of infection, and the host’s immune response (Perfect, 2017). Antifungal activity of MGCD290 is required confirmation using further animal models of IFIs and ultimately in well-designed clinical trials.

## Enolase

Disrupting glycolysis contributes to attenuated fungal virulence (Rutherford et al., 2019). Enolase, also called 2-phospho-D-glycerate hydrolase, is one of the central enzymes of glycolysis, catalyzing the dehydration of 2-phosphoglycerate to phosphoenolpyruvate (Sundstrom and Aliaga, 1994). Enolase (Eno1) has multifunction in fungi and plays a vital role in adhesion, hyphae formation, susceptibility to antifungal drugs, and virulence (Ko et al., 2013). Eno1 is critical for *C. albicans* in the presence of fermentable carbon sources. The homozygous deletion of the *ENO1* gene (*eno1*Δ/Δ) mutant showed higher susceptibility to antifungal drugs, a remarkable reduction in hyphal formation, and a noticeable decrease in pathogenicity in mice models with the wild-type strains (Ko et al., 2013). *A. fumigatus* enolase (AfEno1) expressed at the fungal surface contributes to immune evasion and assists in virulence that can bind the human plasma complement proteins Factor H, FHL-1, C4BP, and plasminogen. Consequently, *A. fumigatus* can damage endothelial cell layers and tissue components (Dasari et al., 2019). Besides, AfEno1 and *C. albicans* enolase (CaEno1) can bind to human plasminogen and then produce plasmin, subsequently improving the invasion and dissemination process during fungal infections (Funk et al., 2016). Furthermore, enolases are conserved among opportunistic pathogenic fungi such as *C. albicans*, *A. fumigatus*, and *C. neoformans*, so inhibitors of fungal enolases will have a broad-antifungal spectrum (Ko et al., 2013).

CaEno1 exists on the outermost layers of the cell wall and is a major antigenic determinant, and the sera of candidemia patients contain high titers Eno1 antibodies (Pitarch et al., 2014). Therefore, exploring monoclonal antibodies against fungal Eno1 is a promising antifungal strategy (Pitarch et al., 2014). Recently, one monoclonal Eno1 antibody MAb R-5 (IgM), inhibited spore germination by 88.3% in *A. fumigatus*, 57.4% in *Aspergillus flavus*, and 30.6% in *A. niger*. It inhibited growth by 24.1%, 13.3%, and 8.8% in *A. fumigatus*, *A. flavus*, and *A. niger*, respectively. Furthermore, Mab R-5 had a protective effect on BALB/c mice challenged intravenously with *A. fumigatus* spores. After being treated with Mab R-5, the colony-forming unit (CFU) of kidney tissue of *A. fumigatus* spores infected mice decreased significantly, and the mean survival time of infected mice was prolonged. These results indicate that MAb R-5 could be valuable in treating IFIs caused by *Aspergillus* species (Yadav and Shukla, 2019). MAb R-5 inhibited spore germination by 88.3% in *A. fumigatus*, 57.4% in *A. flavus*, and 30.6% in *A. niger*. It inhibited growth by 24.1%, 13.3%, and 8.8% in *A. fumigatus*, *A. flavus*, and *A. niger*, respectively. In a prophylactic murine disease model, R-5 reduced *A. fugmigatus* fungal burden by 85.9% and significantly increased survival (Yadav and Shukla, 2019). The MTT reduction assay compared to the control with an irrelevant MAb also confirmed fungicidal activity of MAb R-5, where hyphal damage against *A. fumigatus*, *A. flavus*, and *A. niger* was found to be 24.1%, 13.3%, and 8.8%, respectively.

## Dihydrolactate Dehydrogenase

**Figure 6:**
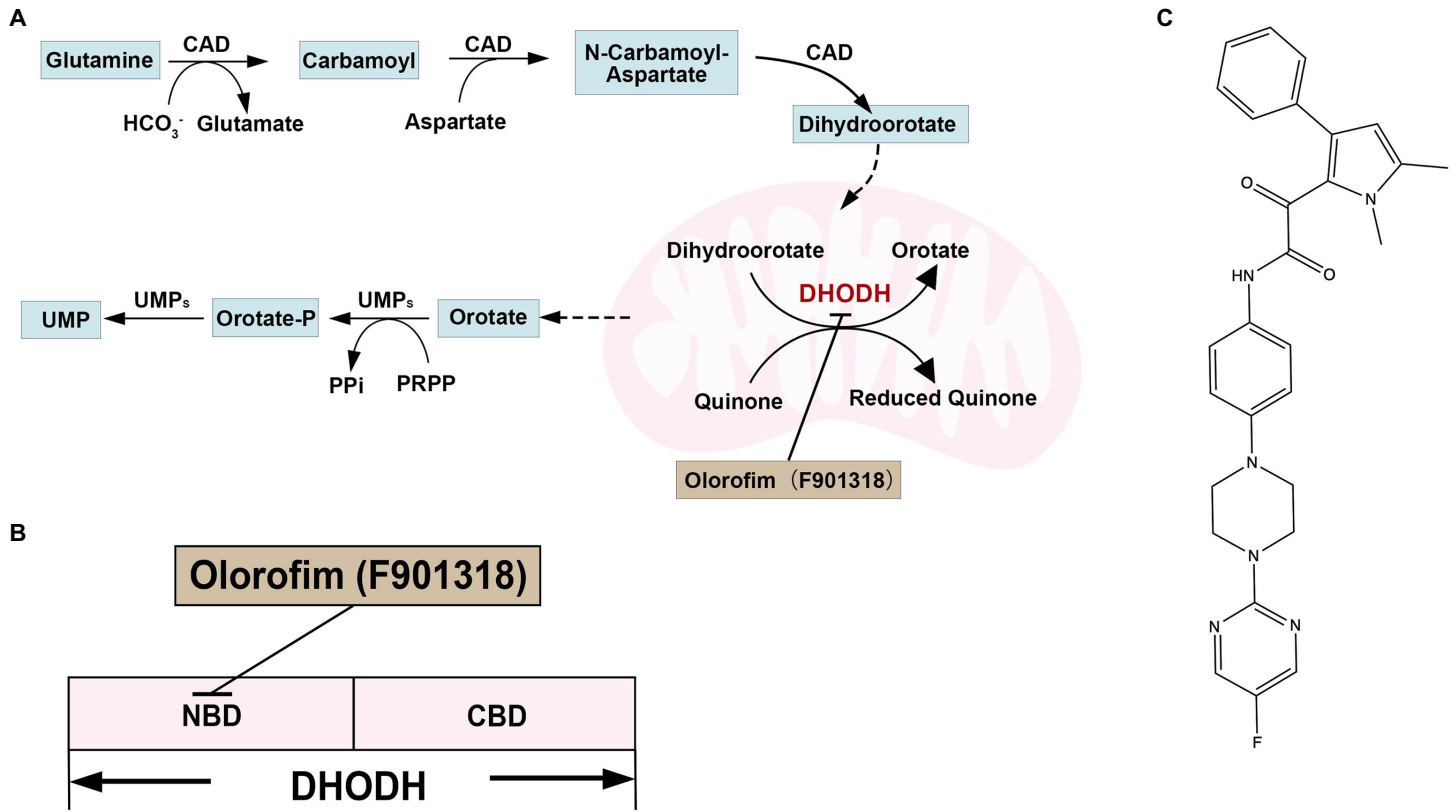
**(A)** Overview of pyrimidine biosynthesis in yeast; **(B)** the docking model of olorofim and DHODH; and **(C)** structure of DHODH inhibitor olorifim (F901318).

Dihydrolactate dehydrogenase (DHODH) contains flavin mononucleotide (FMN) and is the only oxidoreductase among the six enzymes catalyzing the pyrimidine biosynthesis. DHODHs can be divided into two classes based on amino acid sequence similarity, subcellular location, and substrate preference. Furthermore, most pathogens have class II DHODH, which are monomeric proteins and bind to the inner membrane of mitochondria. Class II DHODH contains two structural domains: an N-terminal helix domain consisting of an α/β barrel fold and a C-terminal domain. The N-terminal extension of about 40 residues folds into two α-helices (αA and αB) connected by short loops and responsible for membrane attachment. An additional 20–50 residues exist before the helical structural domain in higher eukaryotes, targeting DHODH to the mitochondrial signal peptide. There are two α helices present in all class II enzymes that form a channel to the active site as a binding site for inhibitors of class II DHODH (Liu et al., 2000; Reis et al., 2017). DHODH catalyzes the fourth step in the pyrimidine biosynthesis pathway, the oxidation of dihydroorotate to orotate, and transfers electrons to coenzyme Q (Figure 6A; Jones, 1980). Pyrimidines are essential for DNA and RNA synthesis and form lipid and carbohydrate metabolism precursors. Therefore, DHODH is a potential antifungal target protein for treating IFIs (Zameitat et al., 2006; Reis et al., 2017; Boschi et al., 2019). Indeed, mutants of *A. fumigatus*, *C. albicans*, and *C. neoformans*, disrupted in pyrimidine biosynthesis, have exhibited attenuated virulence in animal infection models, indicating that targeting pyrimidine synthesis is a promising antifungal strategy (D'Enfert et al., 1996; Noble and Johnson, 2005; de Gontijo et al., 2014). For example, loss of DHODH resulted in a defect in cell wall integrity and increased sensitivity to cell wall-damaging agents and amphotericin B in *C. neoformans* (Banerjee et al., 2016).

Olorofim (F901318; Figure 6C) is a reversible DHODH inhibitor and inhibits DHODH by binding to the N-terminal helical domain of DHODH in *A. fumigatus* (Figure 6B; Oliver et al., 2016). The amino acid identity between *A. fumigatus* DHODH and human DHODH is low (30%; Table 2). Therefore, Olorofim can selectively inhibit the activity of fungal DHODH (Oliver et al., 2016). Although the activity of olorofim against *Candida* species is weak, olorofim has potent antifungal activity against *Aspergillus* species, *Scedosporium* species, *Madurella* species, and *Fusarium* species (Oliver et al., 2016). Furthermore, olorofim has even activity against *A. fumigatus* isolates resistant to other antifungal agents *in vitro* and *vivo* (Du Pré et al., 2018). Olorofim displayed a strong antifungal efficacy in treating aspergillosis in mice caused by azole resistance *Aspergillus* species (Oliver et al., 2016; Du Pré et al., 2018).

In a clinical phase I trial, healthy volunteers orally administrated olorofim once daily (360 mg) for 10 days and well-tolerated olorofim (ClinicalTrials.gov Identifiers: NCT02737371; Kennedy et al., 2017b). This clinical phase I trial showed that plasma concentration of olorofim exceeded the drug exposures required for efficacy in animal models of invasive aspergillosis. No severe AEs were reported, but two participants experienced nausea and diarrhea, and one experienced dizziness. In a double-blind, placebo-controlled, ascending single intravenous dose, sequential group study of 40 healthy male volunteers (ClinicalTrials.gov Identifiers: NCT02142153), there were also no serious AEs reported, with a frequency of nonserious AEs as follows: musculoskeletal pain in 0/30 participants in the olorofim group vs. 1/10 in the placebo group; paresthesias or headache in 2/30 in the olorofim group vs. 0/10 in the placebo group; epistaxis in 2/30 in the olorofim group vs. 1/10 in the placebo group; and eczema in 1/30 participants in the olorofim group vs. 0/10 in the placebo group (Kennedy et al., 2017a). Other two clinical phase I trials of olorofim have also proved the high safety profile of intravenous and oral administration of olorofim (ClinicalTrials.gov Identifiers: NCT02342574, NCT02394483, and NCT03340597), which is currently in the clinical phase II B trials, is used to treat IFIs in patients with limited treatment options (ClinicalTrials.gov identifier: NCT03583164). Currently, a clinical phase III trial to evaluate the efficacy and safety of treatment with olorofim in patients with IFIs caused by *Aspergillus* species is planned (ClinicalTrials.gov Identifier: NCT05101187). In November 2019, the FDA awarded olorofim the title of breakthrough drug treatment. Subsequently, it was awarded the orphan drug title for the treatment of invasive *aspergillosis*, *Lomentospora*/*Scedosporium* infection (March 2020), and *coccidiosis* (June 2020), and recently became a qualified drug for treating infectious diseases (June 2020).

## Conclusions and Perspectives

The increasing prevalence of drug-resistance IFIs and the disadvantages of the current antifungal drugs demand new antifungal drugs (Prasad et al., 2017). In summary, there are several ideas for developing novel antifungal drugs. One strategy is to high-throughput screen compound libraries, small molecule compounds, or natural compound libraries, obtain compounds with remarkable antifungal activity, and then identify their antifungal targets. AbA (targeting IPC synthase), Nikkomycin Z (targeting chitin synthase), Olorofim (targeting DHODH), and Turbinmicin (targeting Sec14) were found in this way. The second way is utilizing a chemical genomics-based screening platform to screen synthetic or natural product libraries and identify target-specific inhibitors with antifungal drug-like properties. M743, an inhibitor of Mcd4, was found in this way. Third, screened candidate compounds specifically target fungal-specific pathways, such as constructing and expressing the small *Gaussia princeps* luciferase gene fused to the GPI-anchoring signal of *C. albicans* cells. Jawsamycin, which targeted Spt14, was discovered using this method. Besides, we can design inhibitors against a specific protein with low toxicity to mammalian cells, such as specific fungal Hsp90 inhibitor CMLD013075 and Gwt1 inhibitor APX001A. The development of biopharmaceuticals is new antifungal therapy that can act as a counterpart in treating IFIs. Monoclonal antibodies, cytokine immunotherapy, vaccines, and antifungal peptides help treat IFIs (Stevens, 1998; Stevens et al., 2000).

New targets for the treatment of IFIs are being developed, but the process has not been as smooth as expected. The following reasons cause this situation: (1) fungal cells are eukaryotic so that potential targets may be conserved in the human host. Such conservation results in compounds having suitable antifungal activities *in vitro* but working poorly *in vivo* because the compounds have toxic side effects. (2) crystal structures of fungal proteins are limited, preventing efficient protein-targeted virtual drug screening and computer-aided drug design. (3) efficient large-scale gene operation studies are challenging in fungi. More potential antifungal targets may be found using CRISPR-Cas9-based gene drive platforms (Shapiro et al., 2018).

## Author Contributions

CZ, HL, and YJ wrote the manuscript draft. HL and YJ conceived the idea. All authors contributed to the article and approved the submitted version.

## Funding

This study was supported by the National Natural Science Foundation of China (no. 82020108032), the Innovation Program of Shanghai Municipal Education Commission (202101070007-E00094), and the National Key Research and Development Program of China (2021YFC2300404).

## Conflict of Interest

The authors declare that the research was conducted in the absence of any commercial or financial relationships that could be construed as a potential conflict of interest.

## Publisher’s Note

All claims expressed in this article are solely those of the authors and do not necessarily represent those of their affiliated organizations, or those of the publisher, the editors and the reviewers. Any product that may be evaluated in this article, or claim that may be made by its manufacturer, is not guaranteed or endorsed by the publisher.

## References

[ref1] Aeed Paul A. Young Casey L. Nagiec M. M. Åke P. E. (2009). Inhibition of inositol phosphorylceramide synthase by the cyclic peptide aureobasidin A. Antimicrob. Agents Chemother. 53, 496–504. doi: 10.1128/AAC.00633-08, PMID: 19047657 PMC2630602

[ref2] Banerjee D. Umland T. C. Panepinto J. C. (2016). *De novo* pyrimidine biosynthesis connects cell integrity to amphotericin B susceptibility in *Cryptococcus neoformans*. mSphere. 1:e00191–16. doi: 10.1128/mSphere.00191-16, PMID: 27904878 PMC5112334

[ref3] Bankaitis V. A. Malehorn D. E. Emr S. D. Greene R. (1989). The *Saccharomyces cerevisiae* SEC14 gene encodes a cytosolic factor that is required for transport of secretory proteins from the yeast Golgi complex. J. Cell Biol. 108, 1271–1281. doi: 10.1083/jcb.108.4.1271, PMID: 2466847 PMC2115512

[ref4] Bankaitis V. A. Mousley C. J. Schaaf G. (2010). The Sec14 superfamily and mechanisms for crosstalk between lipid metabolism and lipid signaling. Trends Biochem. Sci. 35, 150–160. doi: 10.1016/j.tibs.2009.10.008, PMID: 19926291 PMC2834860

[ref5] Becker J. M. Kauffman S. J. Hauser M. Huang L. Lin M. Sillaots S.. (2010). Pathway analysis of *Candida albicans* survival and virulence determinants in a murine infection model. Proc. Natl. Acad. Sci. U. S. A. 107, 22044–22049. doi: 10.1073/pnas.1009845107, PMID: 21135205 PMC3009777

[ref6] Bentz M. L. Nunnally N. Lockhart S. R. Sexton D. J. Berkow E. L. (2021). Antifungal activity of nikkomycin Z against *Candida auris*. J. Antimicrob. Chemother. 76, 1495–1497. doi: 10.1093/jac/dkab052, PMID: 33677578

[ref7] Berkow E. L. Lockhart S. R. (2018). Activity of novel antifungal compound APX001A against a large collection of *Candida auris*. J. Antimicrob. Chemother. 73, 3060–3062. doi: 10.1093/jac/dky302, PMID: 30085167

[ref8] Bien C. M. Chang Y. C. Nes W. D. Kwon-Chung K. J. Espenshade P. J. (2009). *Cryptococcus neoformans* Site-2 protease is required for virulence and survival in the presence of azole drugs. Mol. Microbiol. 74, 672–690. doi: 10.1111/j.1365-2958.2009.06895.x, PMID: 19818023 PMC2917040

[ref001] Blacklock K. Verkhivker G. M. (2014). Computational modeling of allosteric regulation in the hsp90 chaperones: a statistical ensemble analysis of protein structure networks and allosteric communications. PLoS computational biology 10:e1003679. doi: 10.1371/journal.pcbi.1003679 24922508 PMC4055421

[ref9] Boschi D. Pippione A. C. Sainas S. Lolli M. L. (2019). Dihydroorotate dehydrogenase inhibitors in anti-infective drug research. Eur. J. Med. Chem. 183:111681. doi: 10.1016/j.ejmech.2019.111681, PMID: 31557612

[ref10] Brandão F. Esher S. K. Ost K. S. Pianalto K. Nichols C. B. Fernandes L.. (2018). HDAC genes play distinct and redundant roles in *Cryptococcus neoformans* virulence. Sci. Rep. 8:5209. doi: 10.1038/s41598-018-21965-y 29581526 PMC5979944

[ref11] Bugli F. Cacaci M. Martini C. Torelli R. Posteraro B. Sanguinetti M.. (2013). Human monoclonal antibody-based therapy in the treatment of invasive candidiasis. Clin. Dev. Immunol. 2013:403121. doi: 10.1155/2013/403121, PMID: 23878583 PMC3710647

[ref12] Bulawa C. E. Miller D. W. Henry L. K. Becker J. M. (1995). Attenuated virulence of chitin-deficient mutants of *Candida albicans*. Proc. Natl. Acad. Sci. 92, 10570–10574. doi: 10.1073/pnas.92.23.10570, PMID: 7479842 PMC40653

[ref13] Cheng J. Park T. S. Fischl A. S. Ye X. S. (2001). Cell cycle progression and cell polarity require sphingolipid biosynthesis in *aspergillus nidulans*. Mol. Cell. Biol. 21, 6198–6209. doi: 10.1128/MCB.21.18.6198-6209.2001, PMID: 11509663 PMC87337

[ref14] Curwin A. J. Fairn G. D. McMaster C. R. (2009). Phospholipid transfer protein Sec14 is required for trafficking from endosomes and regulates distinct trans-Golgi export pathways*. J. Biol. Chem. 284, 7364–7375. doi: 10.1074/jbc.M808732200, PMID: 19129178 PMC2652273

[ref15] Dasari P. Koleci N. Shopova I. A. Wartenberg D. Beyersdorf N. Dietrich S.. (2019). Enolase from *aspergillus fumigatus* is a moonlighting protein that binds the human plasma complement proteins factor H, FHL-1, C4BP, and plasminogen. Front. Immunol. 10:2573. doi: 10.3389/fimmu.2019.02573, PMID: 31824478 PMC6883375

[ref16] de Gontijo F. A. Pascon R. C. Fernandes L. Machado J. Jr. Alspaugh J. A. Vallim M. A. (2014). The role of the de novo pyrimidine biosynthetic pathway in *Cryptococcus neoformans* high temperature growth and virulence. Fungal Genet. Biol. 70, 12–23. doi: 10.1016/j.fgb.2014.06.003, PMID: 25011011 PMC4286198

[ref17] D'Enfert C. Diaquin M. Delit A. Wuscher N. Debeaupuis J. P. Huerre M.. (1996). Attenuated virulence of uridine-uracil auxotrophs of *aspergillus fumigatus*. Infect. Immun. 64, 4401–4405. doi: 10.1128/iai.64.10.4401-4405.1996, PMID: 8926121 PMC174389

[ref18] Dickson R. C. (1998). Sphingolipid functions in *Saccharomyces Cerevisiae*: comparison to mammals. Annu. Rev. Biochem. 67, 27–48. doi: 10.1146/annurev.biochem.67.1.27, PMID: 9759481

[ref19] Dorfmueller H. C. Ferenbach A. T. Borodkin V. S. van Aalten D. M. F. (2014). A structural and biochemical model of processive chitin synthesis*. J. Biol. Chem. 289, 23020–23028. doi: 10.1074/jbc.M114.563353, PMID: 24942743 PMC4132801

[ref20] Du Pré S. Beckmann N. Almeida M. C. Sibley G. E. M. Law D. Brand A. C.. (2018). Effect of the novel antifungal drug F901318 (olorofim) on growth and viability of *aspergillus fumigatus*. Antimicrob. Agents Chemother. 62:AAC.00231-18. doi: 10.1128/AAC.00231-18 PMC6105813 29891595

[ref21] Fiedler H.-P. Kurth R. Langhärig J. Delzer J. Zähner H. (1982). Nikkomycins: microbial inhibitors of chitin synthase. J. Chem. Technol. Biotechnol. 32, 271–280. doi: 10.1002/jctb.5030320130

[ref22] Fortwendel J. R. Juvvadi P. R. Pinchai N. Perfect B. Z. Alspaugh J. A. Perfect J. R.. (2009). Differential effects of inhibiting chitin and 1,3-{beta}-D-glucan synthesis in ras and calcineurin mutants of *aspergillus fumigatus*. Antimicrob. Agents Chemother. 53, 476–482. doi: 10.1128/AAC.01154-08, PMID: 19015336 PMC2630655

[ref23] Fu Y. Estoppey D. Roggo S. Pistorius D. Fuchs F. Studer C.. (2020). Jawsamycin exhibits *in vivo* antifungal properties by inhibiting Spt14/Gpi3-mediated biosynthesis of glycosylphosphatidylinositol. Nat. Commun. 11:3387. doi: 10.1038/s41467-020-17221-5, PMID: 32636417 PMC7341893

[ref24] Funk J. Schaarschmidt B. Slesiona S. Hallström T. Horn U. Brock M. (2016). The glycolytic enzyme enolase represents a plasminogen-binding protein on the surface of a wide variety of medically important fungal species. Int. J. Med. Microbiol. 306, 59–68. doi: 10.1016/j.ijmm.2015.11.005, PMID: 26679571

[ref25] Gaughran J. P. Lai M. H. Kirsch D. R. Silverman S. J. (1994). Nikkomycin Z is a specific inhibitor of *Saccharomyces cerevisiae* chitin synthase isozyme Chs3 *in vitro* and *in vivo*. J. Bacteriol. 176, 5857–5860. doi: 10.1128/jb.176.18.5857-5860.1994, PMID: 8083179 PMC196793

[ref26] Gaynor E. C. Mondésert G. Grimme S. J. Reed S. I. Orlean P. Emr S. D. (1999). MCD4 encodes a conserved endoplasmic reticulum membrane protein essential for glycosylphosphatidylinositol anchor synthesis in yeast. Mol. Biol. Cell 10, 627–648. doi: 10.1091/mbc.10.3.627, PMID: 10069808 PMC25192

[ref27] Giaever G. Chu A. M. Ni L. Connelly C. Riles L. Veronneau S.. (2002). Functional profiling of the *Saccharomyces cerevisiae* genome. Nature 418, 387–391. doi: 10.1038/nature00935, PMID: 12140549

[ref28] Gow N. A. Robbins P. W. Lester J. W. Brown A. J. Fonzi W. A. Chapman T.. (1994). A hyphal-specific chitin synthase gene (CHS2) is not essential for growth, dimorphism, or virulence of *Candida albicans*. Proc. Natl. Acad. Sci. 91, 6216–6220. doi: 10.1073/pnas.91.13.6216, PMID: 8016141 PMC44169

[ref29] Hasegawa S. Yamada Y. Iwanami N. Nakayama Y. Nakayama H. Iwatani S.. (2019). Identification and functional characterization of *Candida albicans* mannose–ethanolamine phosphotransferase (Mcd4p). Curr. Genet. 65, 1251–1261. doi: 10.1007/s00294-019-00987-7, PMID: 31073667

[ref30] Hashida-Okado T. Ogawa A. Endo M. Yasumoto R. Takesako K. Kato I. (1996). AUR1, a novel gene conferring aureobasidin resistance on *Saccharomyces cerevisiae*: a study of defective morphologies in Aur1p-depleted cells. Mol. Gen. Genet. 251, 236–244. doi: 10.1007/BF02172923, PMID: 8668135

[ref31] Hata K. Horii T. Miyazaki M. Watanabe N.-A. Okubo M. Sonoda J.. (2011). Efficacy of Oral E1210, a new broad-spectrum antifungal with a novel mechanism of action, in murine models of candidiasis, aspergillosis, and fusariosis. Antimicrob. Agents Chemother. 55, 4543–4551. doi: 10.1128/AAC.00366-11, PMID: 21788462 PMC3187015

[ref32] Heidler S. A. Radding J. A. (1995). The AUR1 gene in *Saccharomyces cerevisiae* encodes dominant resistance to the antifungal agent aureobasidin A (LY295337). Antimicrob. Agents Chemother. 39, 2765–2769. doi: 10.1128/AAC.39.12.2765, PMID: 8593016 PMC163026

[ref33] Hnisz D. Majer O. Frohner I. E. Komnenovic V. Kuchler K. (2010). The Set3/Hos2 histone deacetylase complex attenuates cAMP/PKA signaling to regulate morphogenesis and virulence of *Candida albicans*. PLoS Pathog. 6:e1000889. doi: 10.1371/journal.ppat.1000889, PMID: 20485517 PMC2869326

[ref34] Hodges M. Ople E. Shaw K. Mansbach R. Van Marle S. Van Hoogdalem E.. (2017a). Phase 1 study to assess safety, tolerability and pharmacokinetics of single and multiple oral doses of APX001 and to investigate the effect of food on APX001 bioavailability. Open Forum Infect. Dis. 4:S534. doi: 10.1093/ofid/ofx163.1390

[ref35] Hodges M. Ople E. Shaw K. Mansbach R. Van Marle S. Van Hoogdalem E.. (2017b). First-in-human study to assess safety, tolerability and pharmacokinetics of APX001 administered by intravenous infusion to healthy subjects. Open Forum Infect. Dis.:S526.

[ref36] Hohrman K. Gonçalves D. Morano K. A. Johnson J. L. (2021). Disrupting progression of the yeast Hsp90 folding pathway at different transition points results in client-specific maturation defects. Genetics 217:iyab009. doi: 10.1093/genetics/iyab009, PMID: 33789348 PMC8045699

[ref37] Huang D. S. LeBlanc E. V. Shekhar-Guturja T. Robbins N. Krysan D. J. Pizarro J.. (2020). Design and synthesis of fungal-selective resorcylate aminopyrazole Hsp90 inhibitors. J. Med. Chem. 63, 2139–2180. doi: 10.1021/acs.jmedchem.9b00826, PMID: 31513387 PMC7069776

[ref38] Jones M. E. (1980). Pyrimidine nucleotide biosynthesis in animals: genes, enzymes, and regulation of UMP biosynthesis. Annu. Rev. Biochem. 49, 253–279. doi: 10.1146/annurev.bi.49.070180.001345, PMID: 6105839

[ref39] Kennedy T. Allen G. Steiner J. Heep M. Birch M. (2017a). Assessment of the Duration of Infusion on the Tolerability and Repeat Dose Pharmacokinetics of F901318 in Healthy Volunteers. ECCMID: Viena, Austria.

[ref40] Kennedy T. Allen G. Steiner J. Heep M. Oliver J. Sibley G.. (2017b). “Multiple dose pharmacokinetics of an immediate-release tablet formulation of F901318 in healthy male and female subjects.” Proceedings of the 27th European Congress of Clinical Microbiology and Infectious Diseases, 22–25.

[ref41] Ko H.-C. Hsiao T.-Y. Chen C.-T. Yang Y.-L. (2013). *Candida albicans ENO1* null mutants exhibit altered drug susceptibility, hyphal formation, and virulence. J. Microbiol. 51, 345–351. doi: 10.1007/s12275-013-2577-z, PMID: 23812815

[ref42] Kuroda M. Hashida-Okado T. Yasumoto R. Gomi K. Kato I. Takesako K. (1999). An aureobasidin A resistance gene isolated from *aspergillus* is a homolog of yeast AUR1, a gene responsible for inositol phosphorylceramide (IPC) synthase activity. Mol. Gen. Genet. 261, 290–296. doi: 10.1007/s004380050969, PMID: 10102364

[ref43] Kurome T. Inoue T. Takesako K. Kato I. (1998). Syntheses of antifungal aureobasidin A analogs with alkyl chains for structure-activity relationship. J. Antibiot. 51, 359–367. doi: 10.7164/antibiotics.51.359, PMID: 9589073

[ref44] LaFayette S. L. Collins C. Zaas A. K. Schell W. A. Betancourt-Quiroz M. Gunatilaka A. A.. (2010). PKC signaling regulates drug resistance of the fungal pathogen *Candida albicans* via circuitry comprised of Mkc1, calcineurin, and Hsp90. PLoS Pathog. 6:e1001069. doi: 10.1371/journal.ppat.1001069, PMID: 20865172 PMC2928802

[ref45] Lamoth F. Juvvadi P. R. Fortwendel J. R. Steinbach W. J. (2012). Heat shock protein 90 is required for conidiation and cell wall integrity in *aspergillus fumigatus*. Eukaryot. Cell 11, 1324–1332. doi: 10.1128/EC.00032-12, PMID: 22822234 PMC3486032

[ref46] Larwood D. J. (2020). Nikkomycin Z—ready to meet the promise? J. Fungi 6, 261. doi: 10.3390/jof6040261, PMID: 33143248 PMC7712250

[ref47] Lee Y. Puumala E. Robbins N. Cowen L. E. (2021). Antifungal drug resistance: molecular mechanisms in *Candida albicans* and beyond. Chem. Rev. 121, 3390–3411. doi: 10.1021/acs.chemrev.0c00199, PMID: 32441527 PMC8519031

[ref48] Lenardon M. D. Lesiak I. Munro C. A. Gow N. A. R. (2009). Dissection of the *Candida albicans* class I chitin synthase promoters. Mol. Genet. Genomics 281:459. doi: 10.1007/s00438-009-0423-0 19153767 PMC3468743

[ref49] Levine T. P. Wiggins C. A. R. Munro S. (2000). Inositol phosphorylceramide synthase is located in the Golgi apparatus of *Saccharomyces cerevisiae*. Mol. Biol. Cell 11, 2267–2281. doi: 10.1091/mbc.11.7.2267, PMID: 10888667 PMC14918

[ref50] Li R. K. Rinaldi M. G. (1999). *In vitro* antifungal activity of nikkomycin Z in combination with fluconazole or itraconazole. Antimicrob. Agents Chemother. 43, 1401–1405. doi: 10.1128/AAC.43.6.1401, PMID: 10348760 PMC89286

[ref51] Li X. Robbins N. O'Meara T. R. Cowen L. E. (2017). Extensive functional redundancy in the regulation of *Candida albicans* drug resistance and morphogenesis by lysine deacetylases Hos2, Hda1, Rpd3 and Rpd31. Mol. Microbiol. 103, 635–656. doi: 10.1111/mmi.13578, PMID: 27868254 PMC5296215

[ref52] Li H. Zhou H. Luo Y. Ouyang H. Hu H. Jin C. (2007). Glycosylphosphatidylinositol (GPI) anchor is required in *aspergillus fumigatus* for morphogenesis and virulence. Mol. Microbiol. 64, 1014–1027. doi: 10.1111/j.1365-2958.2007.05709.x, PMID: 17501924

[ref53] Liu O. W. Chun C. D. Chow E. D. Chen C. Madhani H. D. Noble S. M. (2008). Systematic genetic analysis of virulence in the human fungal pathogen *Cryptococcus neoformans*. Cell 135, 174–188. doi: 10.1016/j.cell.2008.07.046, PMID: 18854164 PMC2628477

[ref54] Liu S. Neidhardt E. A. Grossman T. H. Ocain T. Clardy J. (2000). Structures of human dihydroorotate dehydrogenase in complex with antiproliferative agents. Structure 8, 25–33. doi: 10.1016/S0969-2126(00)00077-0, PMID: 10673429

[ref55] Louie A. Stein D. S. Zack J. Z. Liu W. Conde H. Fregeau C.. (2011). Dose range evaluation of Mycograb C28Y variant, a human recombinant antibody fragment to heat shock protein 90, in combination with amphotericin B-desoxycholate for treatment of murine systemic candidiasis. Antimicrob. Agents Chemother. 55, 3295–3304. doi: 10.1128/AAC.01324-10, PMID: 21502626 PMC3122395

[ref56] Luberto C. Toffaletti D. L. Wills E. A. Tucker S. C. Casadevall A. Perfect J. R.. (2001). Roles for inositol-phosphoryl ceramide synthase 1 (IPC1) in pathogenesis of *C. neoformans*. Genes Dev. 15, 201–212. doi: 10.1101/gad.856001, PMID: 11157776 PMC312614

[ref58] Mandala S. M. Thornton R. A. Milligan J. Rosenbach M. Garcia-Calvo M. Bull H. G.. (1998). Rustmicin, a potent antifungal agent, inhibits sphingolipid synthesis at inositol phosphoceramide synthase*. J. Biol. Chem. 273, 14942–14949. doi: 10.1074/jbc.273.24.14942, PMID: 9614099

[ref59] Mandala S. M. Thornton R. A. Rosenbach M. Milligan J. Garcia-Calvo M. Bull H. G.. (1997). Khafrefungin, a novel inhibitor of sphingolipid synthesis*. J. Biol. Chem. 272, 32709–32714. doi: 10.1074/jbc.272.51.32709, PMID: 9405490

[ref60] Maneesri J. Azuma M. Sakai Y. Igarashi K. Matsumoto T. Fukuda H.. (2005). Deletion of MCD4 involved in glycosylphosphatidylinositol (GPI) anchor synthesis leads to an increase in β-1,6-glucan level and a decrease in GPI-anchored protein and mannan levels in the cell wall of *Saccharomyces cerevisiae*. J. Biosci. Bioeng. 99, 354–360. doi: 10.1263/jbb.99.354, PMID: 16233801

[ref61] Mann P. A. McLellan C. A. Koseoglu S. Si Q. Kuzmin E. Flattery A.. (2015). Chemical genomics-based antifungal drug discovery: targeting glycosylphosphatidylinositol (GPI) precursor biosynthesis. ACS Infect. Dis. 1, 59–72. doi: 10.1021/id5000212, PMID: 26878058 PMC4739577

[ref62] Matthews R. Burnie J. (1992). The role of hsp90 in fungal infection. Immunol. Today 13, 345–348. doi: 10.1016/0167-5699(92)90169-8, PMID: 1466751

[ref63] Matthews R. C. Rigg G. Hodgetts S. Carter T. Chapman C. Gregory C.. (2003). Preclinical assessment of the efficacy of mycograb, a human recombinant antibody against fungal HSP90. Antimicrob. Agents Chemother. 47, 2208–2216. doi: 10.1128/AAC.47.7.2208-2216.2003, PMID: 12821470 PMC161838

[ref64] Mellado E. Dubreucq G. Mol P. Sarfati J. Paris S. Diaquin M.. (2003). Cell wall biogenesis in a double chitin synthase mutant (chsG−/chsE−) of *aspergillus fumigatus*. Fungal Genet. Biol. 38, 98–109. doi: 10.1016/S1087-1845(02)00516-9, PMID: 12553940

[ref65] Miyazaki M. Horii T. Hata K. Watanabe N. A. Nakamoto K. Tanaka K.. (2011). *In vitro* activity of E1210, a novel antifungal, against clinically important yeasts and molds. Antimicrob. Agents Chemother. 55, 4652–4658. doi: 10.1128/AAC.00291-11, PMID: 21825291 PMC3186989

[ref66] Monteoliva L. Sánchez M. Pla J. Gil C. Nombela C. (1996). Cloning of *Candida albicans* SEC14 gene homologue coding for a putative essential function. Yeast 12, 1097–1105. doi: 10.1002/(SICI)1097-0061(19960915)12:11<1097::AID-YEA990>3.0.CO;2-E, PMID: 8896277

[ref67] Morozov A. A. Likhoshway Y. V. (2016). Evolutionary history of the chitin synthases of eukaryotes. Glycobiology 26, 635–639. doi: 10.1093/glycob/cww018, PMID: 26887391

[ref68] Munro C. A. Winter K. Buchan A. Henry K. Becker J. M. Brown A. J. P.. (2001). Chs1 of *Candida albicans* is an essential chitin synthase required for synthesis of the septum and for cell integrity. Mol. Microbiol. 39, 1414–1426. doi: 10.1046/j.1365-2958.2001.02347.x, PMID: 11251855

[ref69] Munusamy K. Vadivelu J. Tay S. T. (2018). A study on *Candida* biofilm growth characteristics and its susceptibility to aureobasidin A. Rev. Iberoam. Micol. 35, 68–72. doi: 10.1016/j.riam.2017.07.001, PMID: 29544734

[ref70] Nagiec M. M. Nagiec E. E. Baltisberger J. A. Wells G. B. Lester R. L. Dickson R. C. (1997). Sphingolipid synthesis as a target for antifungal drugs. J. Biol. Chem. 272, 9809–9817. doi: 10.1074/jbc.272.15.9809, PMID: 9092515

[ref71] Nakamura M. Mori Y. Okuyama K. Tanikawa K. Yasuda S. Hanada K.. (2003). Chemistry and biology of khafrefungin. Large-scale synthesis, design, and structure-activity relationship of khafrefungin, an antifungal agent. Org. Biomol. Chem. 1, 3362–3376. doi: 10.1039/B305818B, PMID: 14584800

[ref72] Nicola A. M. Albuquerque P. Paes H. C. Fernandes L. Costa F. F. Kioshima E. S.. (2019). Antifungal drugs: new insights in research & development. Pharmacol. Ther. 195, 21–38. doi: 10.1016/j.pharmthera.2018.10.008, PMID: 30347212

[ref73] Nile A. H. Tripathi A. Yuan P. Mousley C. J. Suresh S. Wallace I. M.. (2014). PITPs as targets for selectively interfering with phosphoinositide signaling in cells. Nat. Chem. Biol. 10, 76–84. doi: 10.1038/nchembio.1389, PMID: 24292071 PMC4059020

[ref74] Nix D. E. Swezey R. R. Hector R. Galgiani J. N. (2009). Pharmacokinetics of nikkomycin Z after single rising oral doses. Antimicrob. Agents Chemother. 53, 2517–2521. doi: 10.1128/AAC.01609-08, PMID: 19349517 PMC2687243

[ref75] Nobile C. J. Mitchell A. P. (2005). Regulation of cell-surface genes and biofilm formation by the *C. albicans* transcription factor Bcr1p. Curr. Biol. 15, 1150–1155. doi: 10.1016/j.cub.2005.05.047, PMID: 15964282

[ref76] Noble S. M. Johnson A. D. (2005). Strains and strategies for large-scale gene deletion studies of the diploid human fungal pathogen *Candida albicans*. Eukaryot. Cell 4, 298–309. doi: 10.1128/EC.4.2.298-309.2005, PMID: 15701792 PMC549318

[ref77] Nooney L. Al-Akeel R. Awad S. AlShami I. Matthews R. Burnie J. (2007). Oral presentations. Int. J. Antimicrob. Agents 29, S565–S566. doi: 10.1016/S0924-8579(07)71806-X, PMID: 35647310

[ref78] Oliver J. D. Sibley G. E. M. Beckmann N. Dobb K. S. Slater M. J. Mcentee L.. (2016). F901318 represents a novel class of antifungal drug that inhibits dihydroorotate dehydrogenase. Proc. Natl. Acad. Sci. 113, 12809–12814. doi: 10.1073/pnas.1608304113, PMID: 27791100 PMC5111691

[ref79] Perfect J. R. (2017). The antifungal pipeline: a reality check. Nat. Rev. Drug Discov. 16, 603–616. doi: 10.1038/nrd.2017.46, PMID: 28496146 PMC5760994

[ref80] Perlin D. S. (2015). Mechanisms of echinocandin antifungal drug resistance. Ann. N. Y. Acad. Sci. 1354, 1–11. doi: 10.1111/nyas.12831, PMID: 26190298 PMC4626328

[ref81] Peter O. Menon A. K. (2007). Thematic review series: lipid posttranslational modifications. GPI anchoring of protein in yeast and mammalian cells, or: how we learned to stop worrying and love glycophospholipids. J. Lipid Res. 48, 993–1011. doi: 10.1194/jlr.R700002-JLR200, PMID: 17361015

[ref82] Pfaller M. A. Diekema D. J. Turnidge J. D. Castanheira M. Jones R. N. (2019). Twenty years of the SENTRY antifungal surveillance program: results for *Candida* species from 1997–2016. Open Forum Infect. Dis. 6, S79–S94. doi: 10.1093/ofid/ofy358, PMID: 30895218 PMC6419901

[ref83] Pfaller M. A. Messer S. A. Georgopapadakou N. Martell L. A. Besterman J. M. Diekema D. J. (2009). Activity of MGCD290, a Hos2 histone deacetylase inhibitor, in combination with azole antifungals against opportunistic fungal pathogens. J. Clin. Microbiol. 47, 3797–3804. doi: 10.1128/JCM.00618-09, PMID: 19794038 PMC2786684

[ref84] Pfaller M. A. Rhomberg P. R. Messer S. A. Castanheira M. (2015). *In vitro* activity of a Hos2 deacetylase inhibitor, MGCD290, in combination with echinocandins against echinocandin-resistant *Candida* species. Diagn. Microbiol. Infect. Dis. 81, 259–263. doi: 10.1016/j.diagmicrobio.2014.11.008, PMID: 25600842

[ref85] Pijnappel W. W. Schaft D. Roguev A. Shevchenko A. Tekotte H. Wilm M.. (2001). The *S. cerevisiae* SET3 complex includes two histone deacetylases, Hos2 and Hst1, and is a meiotic-specific repressor of the sporulation gene program. Genes Dev. 15, 2991–3004. doi: 10.1101/gad.207401, PMID: 11711434 PMC312828

[ref86] Pitarch A. Nombela C. Gil C. (2014). Serum antibody signature directed against *Candida albicans* Hsp90 and enolase detects invasive candidiasis in non-neutropenic patients. J. Proteome Res. 13, 5165–5184. doi: 10.1021/pr500681x, PMID: 25377742

[ref87] Pittet M. Conzelmann A. (2007). Biosynthesis and function of GPI proteins in the yeast *Saccharomyces cerevisiae*. Biochim. Biophys. Acta Mol. Cell Biol. Lipids 1771, 405–420. doi: 10.1016/j.bbalip.2006.05.015, PMID: 16859984

[ref88] Prasad R. Banerjee A. Shah A. H. (2017). Resistance to antifungal therapies. Essays Biochem. 61, 157–166. doi: 10.1042/EBC20160067, PMID: 28258238

[ref89] Reis R. A. G. Calil F. A. Feliciano P. R. Pinheiro M. P. Nonato M. C. (2017). The dihydroorotate dehydrogenases: past and present. Arch. Biochem. Biophys. 632, 175–191. doi: 10.1016/j.abb.2017.06.019, PMID: 28666740

[ref90] Robbins N. Uppuluri P. Nett J. Rajendran R. Ramage G. Lopez-Ribot J. L.. (2011). Hsp90 governs dispersion and drug resistance of fungal biofilms. PLoS Pathog. 7:e1002257. doi: 10.1371/journal.ppat.1002257, PMID: 21931556 PMC3169563

[ref91] Robbins N. Wright G. D. Cowen L. E. (2016). Antifungal drugs: the current armamentarium and development of new agents. Microbiol. Spectr. 4. doi: 10.1128/microbiolspec.FUNK-0002-2016, PMID: 27763259

[ref92] Rutherford J. C. Bahn Y. S. van den Berg B. Heitman J. Xue C. (2019). Nutrient and stress sensing in pathogenic yeasts. Front. Microbiol. 10:442. doi: 10.3389/fmicb.2019.00442, PMID: 30930866 PMC6423903

[ref93] Sagane K. Umemura M. Ogawa-Mitsuhashi K. Tsukahara K. Yoko-o T. Jigami Y. (2011). Analysis of membrane topology and identification of essential residues for the yeast endoplasmic reticulum inositol acyltransferase Gwt1p. J. Biol. Chem. 286, 14649–14658. doi: 10.1074/jbc.M110.193490, PMID: 21367863 PMC3077662

[ref94] Saito K. Tautz L. Mustelin T. (2007). The lipid-binding SEC14 domain. Biochim. Biophys. Acta Mol. Cell Biol. Lipids 1771, 719–726. doi: 10.1016/j.bbalip.2007.02.010, PMID: 17428729

[ref95] Schönbächler M. Horvath A. Fassler J. Riezman H. (1995). The yeast spt14 gene is homologous to the human PIG-A gene and is required for GPI anchor synthesis. EMBO J. 14, 1637–1645. doi: 10.1002/j.1460-2075.1995.tb07152.x, PMID: 7737116 PMC398256

[ref96] Scroggins B. T. Robzyk K. Wang D. Marcu M. G. Tsutsumi S. Beebe K.. (2007). An acetylation site in the middle domain of Hsp90 regulates chaperone function. Mol. Cell 25, 151–159. doi: 10.1016/j.molcel.2006.12.008, PMID: 17218278 PMC1839984

[ref97] Segal E. S. Gritsenko V. Levitan A. Yadav B. Dror N. Steenwyk J. L.. (2018). Gene essentiality analyzed by *in vivo* transposon mutagenesis and machine learning in a stable haploid isolate of *Candida albicans*. MBio 9, e02048–e020418. doi: 10.1128/mBio.02048-18 30377286 PMC6212825

[ref98] Shapiro R. S. Chavez A. Porter C. B. M. Hamblin M. Kaas C. S. DiCarlo J. E.. (2018). A CRISPR-Cas9-based gene drive platform for genetic interaction analysis in *Candida albicans*. Nat. Microbiol. 3, 73–82. doi: 10.1038/s41564-017-0043-0, PMID: 29062088 PMC5832965

[ref99] Stevens D. A. (1998). Combination immunotherapy and antifungal chemotherapy. Clin. Infect. Dis. 26, 1266–1269. doi: 10.1086/516362, PMID: 9636844

[ref100] Stevens D. A. Kullberg B. J. Brummer E. Casadevall A. Netea M. G. Sugar A. M. (2000). Combined treatment: antifungal drugs with antibodies, cytokines or drugs. Med. Mycol. 38, 305–315. doi: 10.1080/mmy.38.s1.305.315 11204158

[ref101] Sundstrom P. Aliaga G. R. (1994). A subset of proteins found in culture supernatants of *Candida albicans* includes the abundant, immunodominant, glycolytic enzyme enolase. J Infect Dis 169, 452–456. doi: 10.1093/infdis/169.2.452, PMID: 8106783

[ref102] Takesako K. Kuroda H. Inoue T. Haruna F. Yoshikawa Y. Kato I.. (1993). Biological properties of aureobasidin A, a cyclic depsipeptide antifungal antibiotic. J. Antibiot. 46, 1414–1420. doi: 10.7164/antibiotics.46.1414, PMID: 8226319

[ref103] Tan H. W. Tay S. T. (2013). The inhibitory effects of aureobasidin A on *Candida* planktonic and biofilm cells. Mycoses 56, 150–156. doi: 10.1111/j.1439-0507.2012.02225.x, PMID: 22882276

[ref104] Teymuri M. Shams-Ghahfarokhi M. Razzaghi-Abyaneh M. (2021). Inhibitory effects and mechanism of antifungal action of the natural cyclic depsipeptide, aureobasidin A against *Cryptococcus neoformans*. Bioorg. Med. Chem. Lett. 41:128013. doi: 10.1016/j.bmcl.2021.128013, PMID: 33811994

[ref105] Thiagalingam S. Cheng K. H. Lee H. J. Mineva N. Thiagalingam A. Ponte J. F. (2003). Histone deacetylases: unique players in shaping the epigenetic histone code. Ann. N. Y. Acad. Sci. 983, 84–100. doi: 10.1111/j.1749-6632.2003.tb05964.x, PMID: 12724214

[ref106] Tsukahara K. Hata K. Nakamoto K. Sagane K. Watanabe N.-A. Kuromitsu J.. (2003). Medicinal genetics approach towards identifying the molecular target of a novel inhibitor of fungal cell wall assembly. Mol. Microbiol. 48, 1029–1042. doi: 10.1046/j.1365-2958.2003.03481.x, PMID: 12753194

[ref107] Umemura M. Okamoto M. Nakayama K. Sagane K. Tsukahara K. Hata K.. (2003). GWT1 gene is required for inositol acylation of glycosylphosphatidylinositol anchors in yeast. J. Biol. Chem. 278, 23639–23647. doi: 10.1074/jbc.M301044200, PMID: 12714589

[ref108] Van Daele R. Spriet I. Wauters J. Maertens J. Mercier T. Van Hecke S.. (2019). Antifungal drugs: what brings the future? Med. Mycol. 57, S328–S343. doi: 10.1093/mmy/myz012, PMID: 31292663

[ref109] van der Rest M. E. Kamminga A. H. Nakano A. Anraku Y. Poolman B. Konings W. N. (1995). The plasma membrane of *Saccharomyces cerevisiae*: structure, function, and biogenesis. Microbiol. Rev. 59, 304–322. doi: 10.1128/mr.59.2.304-322.1995, PMID: 7603412 PMC239363

[ref110] Vu K. Tham R. Uhrig J. P. Thompson G. R. 3rd Na Pombejra S. Jamklang M.. (2014). Invasion of the central nervous system by *Cryptococcus neoformans* requires a secreted fungal metalloprotease. MBio 5, e01101–e01114. doi: 10.1128/mBio.01101-14, PMID: 24895304 PMC4049100

[ref111] Wakabayashi T. Mori K. Kobayashi S. (2001). Total synthesis and structural elucidation of khafrefungin. J. Am. Chem. Soc. 123, 1372–1375. doi: 10.1021/ja0057272, PMID: 11456709

[ref112] Walker L. A. Munro C. A. de Bruijn I. Lenardon M. D. McKinnon A. Gow N. A. (2008). Stimulation of chitin synthesis rescues *Candida albicans* from echinocandins. PLoS Pathog. 4:e1000040. doi: 10.1371/journal.ppat.1000040, PMID: 18389063 PMC2271054

[ref113] Watanabe N. A. Miyazaki M. Horii T. Sagane K. Tsukahara K. Hata K. (2012). E1210, a new broad-spectrum antifungal, suppresses *Candida albicans* hyphal growth through inhibition of glycosylphosphatidylinositol biosynthesis. Antimicrob. Agents Chemother. 56, 960–971. doi: 10.1128/AAC.00731-11, PMID: 22143530 PMC3264227

[ref114] Whitesell L. Robbins N. Huang D. S. McLellan C. A. Shekhar-Guturja T. LeBlanc E. V.. (2019). Structural basis for species-selective targeting of Hsp90 in a pathogenic fungus. Nat. Commun. 10:402. doi: 10.1038/s41467-018-08248-w, PMID: 30679438 PMC6345968

[ref115] Wiederhold N. P. Najvar L. K. Shaw K. J. Jaramillo R. Patterson H. Olivo M.. (2019). Efficacy of delayed therapy with fosmanogepix (APX001) in a murine model of *Candida auris* invasive candidiasis. Antimicrob. Agents Chemother. 63, e01120–e01119. doi: 10.1128/AAC.01120-19 31427304 PMC6811405

[ref116] Wuts P. G. M. Simons L. J. Metzger B. P. Sterling R. C. Slightom J. L. Elhammer A. P. (2015). Generation of broad-spectrum antifungal drug candidates from the natural product compound aureobasidin A. ACS Med. Chem. Lett. 6, 645–649. doi: 10.1021/acsmedchemlett.5b00029, PMID: 26101567 PMC4468416

[ref117] Xu D. Jiang B. Ketela T. Lemieux S. Veillette K. Martel N.. (2007). Genome-wide fitness test and mechanism-of-action studies of inhibitory compounds in *Candida albicans*. PLoS Pathog. 3:e92. doi: 10.1371/journal.ppat.0030092, PMID: 17604452 PMC1904411

[ref118] Yadav U. Khan M. A. (2018). Targeting the GPI biosynthetic pathway. Pathog. Glob. Health 112, 115–122. doi: 10.1080/20477724.2018.1442764, PMID: 29484956 PMC6056829

[ref119] Yadav R. K. Shukla P. K. (2019). A novel monoclonal antibody against enolase antigen of *aspergillus fumigatus* protects experimental aspergillosis in mice. FEMS Microbiol. Lett. 366:fnz015. doi: 10.1093/femsle/fnz015, PMID: 30649286

[ref120] Yang J. Zhang K.-Q. (2019). “Chitin synthesis and degradation in fungi: biology and enzymes,” in Targeting Chitin-Containing Organisms. eds. Yang Q. Fukamizo T. (Singapore: Springer Singapore), 153–167. 10.1007/978-981-13-7318-3_8 31102246

[ref121] Yano T. Aoyagi A. Kozuma S. Kawamura Y. Tanaka I. Suzuki Y.. (2007). Pleofungins, novel inositol phosphorylceramide synthase inhibitors, from *Phoma* sp. SANK 13899. J. Antibiot. 60, 136–142. doi: 10.1038/ja.2007.13, PMID: 17420564

[ref122] Yoshida M. Ezaki M. Hashimoto M. Yamashita M. Shigematsu N. Okuhara M.. (1990). A novel antifungal antibiotic, FR-900848. I. Production, isolation, physico-chemical and biological properties. J. Antibiot. 43, 748–754. doi: 10.7164/antibiotics.43.748, PMID: 2387768

[ref123] Zacchi L. F. Schulz W. L. Davis D. A. (2010). HOS2 and HDA1 encode histone deacetylases with opposing roles in *Candida albicans* morphogenesis. PLoS One 5:e12171. doi: 10.1371/journal.pone.0012171, PMID: 20730094 PMC2921335

[ref124] Zameitat E. Gojković Z. Knecht W. Piškur J. Löffler M. (2006). Biochemical characterization of recombinant dihydroorotate dehydrogenase from the opportunistic pathogenic yeast *Candida albicans*. FEBS J. 273, 3183–3191. doi: 10.1111/j.1742-4658.2006.05327.x, PMID: 16774642

[ref125] Zavrel M. White T. C. (2015). Medically important fungi respond to azole drugs: an update. Future Microbiol. 10, 1355–1373. doi: 10.2217/FMB.15.47, PMID: 26234644

[ref126] Zhang F. Zhao M. Braun D. R. Ericksen S. S. Piotrowski J. S. Nelson J.. (2020). A marine microbiome antifungal targets urgent-threat drug-resistant fungi. Science 370, 974–978. doi: 10.1126/science.abd6919, PMID: 33214279 PMC7756952

[ref127] Zhao M. Zhang F. Zarnowski R. Barns K. J. Jones R. Fossen J. L.. (2020). Turbinmicin inhibits *Candida* biofilm growth by disrupting fungal vesicle-mediated trafficking. J. Clin. Investig. 131:e145123. doi: 10.1172/JCI145123 PMC7919718 33373326

